# Enhanced Surgical Decision-Making Tools in Breast Cancer: Predicting 2-Year Postoperative Physical, Sexual, and Psychosocial Well-Being following Mastectomy and Breast Reconstruction (INSPiRED 004)

**DOI:** 10.1245/s10434-023-13971-w

**Published:** 2023-07-30

**Authors:** Cai Xu, André Pfob, Babak J. Mehrara, Peimeng Yin, Jonas A. Nelson, Andrea L. Pusic, Chris Sidey-Gibbons

**Affiliations:** 1 Section of Patient Centered Analytics, Division of Internal Medicine, The University of Texas MD Anderson Cancer Center, Houston, TX USA; 2 MD Anderson Center for INSPiRED Cancer Care (Integrated Systems for Patient-Reported Data), The University of Texas MD Anderson Cancer Center, Houston, TX USA; 3 Department of Obstetrics and Gynecology, Heidelberg University Hospital, Heidelberg, Germany; 4 Department of Plastic and Reconstructive Surgery, Memorial Sloan Kettering Cancer Center, New York, NY USA; 5 Computer Science and Mathematics Division, Oak Ridge National Laboratory, Oak Ridge, TN USA; 6 Department of Surgery, Patient-Reported Outcome Value and Experience (PROVE) Center, Harvard Medical School & Brigham and Women’s Hospital, Boston, MA USA

**Keywords:** Machine learning, Postmastectomy breast reconstruction, PRO, QOL

## Abstract

**Background:**

We sought to predict clinically meaningful changes in physical, sexual, and psychosocial well-being for women undergoing cancer-related mastectomy and breast reconstruction 2 years after surgery using machine learning (ML) algorithms trained on clinical and patient-reported outcomes data.

**Patients and Methods:**

We used data from women undergoing mastectomy and reconstruction at 11 study sites in North America to develop three distinct ML models. We used data of ten sites to predict clinically meaningful improvement or worsening by comparing pre-surgical scores with 2 year follow-up data measured by validated Breast-Q domains. We employed ten-fold cross-validation to train and test the algorithms, and then externally validated them using the 11th site’s data. We considered area-under-the-receiver-operating-characteristics-curve (AUC) as the primary metric to evaluate performance.

**Results:**

Overall, between 1454 and 1538 patients completed 2 year follow-up with data for physical, sexual, and psychosocial well-being. In the hold-out validation set, our ML algorithms were able to predict clinically significant changes in physical well-being (chest and upper body) (worsened: AUC range 0.69–0.70; improved: AUC range 0.81–0.82), sexual well-being (worsened: AUC range 0.76–0.77; improved: AUC range 0.74–0.76), and psychosocial well-being (worsened: AUC range 0.64–0.66; improved: AUC range 0.66–0.66). Baseline patient-reported outcome (PRO) variables showed the largest influence on model predictions.

**Conclusions:**

Machine learning can predict long-term individual PROs of patients undergoing postmastectomy breast reconstruction with acceptable accuracy. This may better help patients and clinicians make informed decisions regarding expected long-term effect of treatment, facilitate patient-centered care, and ultimately improve postoperative health-related quality of life.

**Supplementary Information:**

The online version contains supplementary material available at 10.1245/s10434-023-13971-w.

Postmastectomy breast reconstruction (PMBR) has important long-term effects on quality of life (QOL).^1^ With advances in reconstructive techniques and an increasing number of women undergoing risk reducing mastectomy, there is a trend toward a rising demand of PMBR.^2^ PBMR is beneficial for improving body image and minimizing the negative impact of mastectomy on QOL.^3^ However, facing different breast reconstruction treatment options (e.g., implant-based versus autologous), many women have difficulties making high-quality decisions due to anecdotal methods used for patient education.^4^

Clinical studies have been conducted to compare different options and evaluate the outcome of PMBR, to provide insights into treatment options, and to inform patients’ decision-making.^5–7^ For example, a previous prospective cohort study concluded that autologous reconstruction offers benefits over implant-based reconstruction in terms of QOL.^1^ However, recommendations and conclusions derived from group-level studies are not suitable for a specific individual’s situation. Tailoring individual care to match each patient’s expected QOL after reconstruction is necessary and warrants further investigation. Fortunately, the emergence of cutting-edge computational techniques—machine learning (ML)—accompanied by the usage of individual patient-reported outcome (PRO) data provides the potential to address this knowledge gap and to help patients and clinicians make informed decisions before the initiation of breast reconstruction procedures to facilitate patient-centered care.

As a branch of artificial intelligence, ML involves training algorithms to identify intricate patterns within data and make precise predictions.^8^ By learning patterns from data, ML has the unique capability to predict future outcomes at the individual level. This ability to provide personalized predictions and recommendations tailored to individual patients has the potential to greatly enhance patient care, leading to growing enthusiasm for the application of ML techniques in addressing clinical problems. Trained ML models using supervised learning techniques have consistently demonstrated exceptional performance across a range of challenging prediction tasks in the medical field. These tasks include, but are not limited to, prediction of mortality in cancer patients,^9^ natural language processing,^10^ prediction of financial toxicity caused by cancer treatment,^11^ and classification of benign or malignant tumor.^12^ The success of ML in these predictive tasks can be attributed to its strong capability to identify subtle nonlinear interactions between events and outcomes within multidimensional data.^13^ This ability allows ML models to uncover complex relationships that may not be discernible through traditional methods, resulting in more accurate predictions and improved decision-making across various healthcare domains.

Machine learning algorithms have previously achieved excellent performance in predicting breast satisfaction, one of the key outcomes for women undergoing PMBR, both at 1 and 2 year follow-up.^14,15^ In this comprehensive study, we aimed to develop and validate ML algorithms to accurately predict clinically meaningful, long-term changes in physical, sexual, and psychosocial well-being for women undergoing PMBR at 2 year follow-up to enhance decision-making in this area, shifting a focus from satisfaction to the critical areas of health-related QOL, using the same study population as those prior two studies, and affording unique insights into the PRO on the health-related QOL prediction for women with breast cancer.

## Patients and Methods

### Study Participants

This study cohort was a subgroup of the international Mastectomy Reconstruction Outcomes Consortium (MROC, NCT01723423) study that was conducted at 11 study sites in both Canada and the USA between 2012 and 2017. A total of 3058 women undergoing PMBR were recruited as described in detail elsewhere.^1,14^

Inclusion criteria were women aged 18 years or older, undergoing first time bilateral or unilateral, immediate or delayed PMBR for risk reducing or therapeutic purposes. These patients could have undergone implant-based and/or autologous reconstruction, based on the surgeon’s recommendation or their preferences. Exclusion criteria were patients with previous failed breast reconstruction. For the present analysis, patients with unreported PROs at baseline or 2 year follow-up were also excluded.

All included study sites received ethical approval from the respective institutional review board.

### Study Design

Patient-reported physical, sexual, and psychosocial well-being were evaluated before the initiation of the reconstruction procedure and at 2 year follow up by the validated and reliable BREAST-Q.^16^ Cronbach’s alpha coefficients are reportedly greater than 0.8 and the score of each scale ranges from 0 (worst well-being) to 100 (best well-being).^17^

Minimal clinically important difference (MCID) estimates have previously been reported: MCID in physical well-being (chest and upper body) is a score difference of at least 3, and a score difference of at least 4 in both sexual and psychosocial well-being.^18^ We defined three types of outcomes for each domain when comparing baseline PROs with those at 2 year follow-up: outcomes of health-related QOL were (1) worsened if the 2-year follow-up score was reduced at least by the respective MCIDs compared to baseline, (2) improved if the 2-year follow-up score was increased at least by the respective MCIDs compared with baseline, or (3) otherwise stable.

To facilitate the construction of ML predictive models, we recoded the outcome into binary (i.e., improved versus not improved, worsened versus not worsened).

### Algorithm Selection

We trained three ML algorithms with varying levels of complexity for each domain given their demonstrated promising performance in published similar medical studies conducted by our team,^14,15,19^ and reported findings following relevant guidelines (TRIPOD).^20^

We briefly describe each algorithm below. A detailed description can be found in online supplemental documents of our previously published study.^14^

1. Logistic regression (LR) with elastic net penalty.
  The LR with penalized magnitudes of coefficients is known for its easy-to-interpret prediction process, ability to avoid overfitting, and enhanced generalizability on new datasets.^13^
2. Extreme gradient boosting (XGBoost) tree.
  The XGboost tree, as an ensemble-learning algorithm of several built models, is suitable for complex classification tasks due to its enhanced capability in identifying complex relationships among predictors.^21^
3. Neural network.
  A neural network has a unique network structure consisting of connected units that is inspired by the structure of the human cortex. This enables identification of complex patterns within the dataset and capturing nonlinear relations among the input and output variables.

### Data Preparation

We split the 11-site data into a development set of 10 sites and a validation set of 1 site. The validation site with initials of “BW” was chosen based on the number of events, as reported in our previous research on breast satisfaction prediction.^14^ We included four patients, five preoperative PRO, and seven clinical variables as predictive factors (Table 1 in Supplement 1).

For data preparation, we imputed missing values using the K-nearest neighbors algorithm (*K* = 5), removed zero variance variables, centered and scaled all numerical variables, and dummied all categorical variables with one hot encoding. Variables having an absolute correlation with other variables over a threshold of 0.9 were removed, to address the multicollinearity issues.

For ML algorithm training and internal testing on the development set, we adopted ten-fold cross-validation with three repetitions and a hypergrid search to train the models and tune hyperparameters. We computed sensitivity, specificity, the area-under-the-receiver-operating-characteristics-curve (AUC), precision, and recall, to assess model performance in each fold. We embraced the “Kappa” metric to evaluate final model performance in the test fold because of the possible class-imbalance effect. We chose the simplest model that was within a 3% tolerance of the empirically optimal model as the final model to reduce overfitting and improve generalizability to new datasets.^22^

Based on our previous research, we excluded five socioeconomic and racial variables to avoid racial bias.^23^ We compared model performance among each racial group to evaluate the fairness of ML algorithms.^24^

### Analysis Strategies

The predictive performance of the ML algorithms were measured via accuracy and AUC. Point estimates along with a 95% confidence interval (CI) are reported. To provide insights into model predictions and improve transparency and interpretability, we reported regularized coefficients for the LR with elastic net penalty, Shapley Additive explanations (SHAP) values for XGBoost tree,^25^ and local interpretable model-agnostic explanations (LIME) for the neural network.^26^ For comparison, traditional binary logistic regression models are provided as well. To assess the fairness of model performance, we compared the predictive performance of the models across all racial groups in the validation set. To assess algorithm calibration in the validation set, we plot calibration plots with predicted versus observed rates of outcome.^27^ We conducted the Spiegelhalter Z test for calibration accuracy assessment,^28^ with a *p*-value greater than 0.05 indicating the model was well calibrated. We calculated the scaled Brier score with a range between 0 (perfect predictive performance) and 1 (poor predictive performance).^29^

Lastly, we conduct receiver operating characteristic curve comparisons among ML model performance in both development and validation sets for each scale to assess their statistical significance. We plotted AUC of models to predict improved and worsened health-related QOL outcomes together to get the full picture of the performance of the trained ML model at each scale.

We carried out all analyses within the “R” programming environment with version 4.2.1. and developed ML models using the “caret” package.

## Results

### Clinical and Demographic Characteristics

**Fig. 1:**
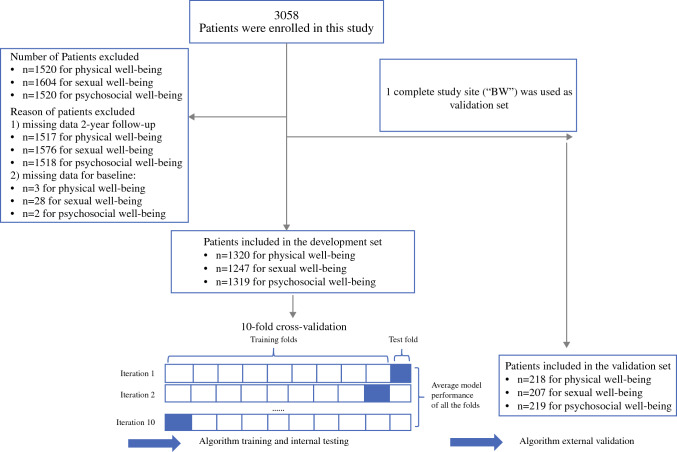
Study design and flow of participants

The analysis set comprised 1538 participants for physical well-being (1320 development and 218 validation), 1454 for sexual well-being (1247 development and 207 validation), and 1538 for psychosocial well-being (1319 development and 219 validation) to train and validate ML models as shown in Fig. 1. The baseline demographic and clinical characteristics for all three BREAST-Q scales are presented in Table 1 (Table 1 with details in Supplement 2). Two years after breast reconstruction, 563 (36.6%) patients experienced improved physical well-being, 592 (40.7%) improved sexual well-being, and 769 (50.0%) improved psychosocial well-being, whereas 737 (47.9%), 647 (44.5%), and 453 (29.5%) patients experienced worsening in physical, sexual, and psychosocial well-being 2 years after surgery, respectively.

**Table 1:** Participant baseline characteristics and health outcomes

	Physical well-being (chest and upper body)			Sexual well-being			Psychosocial well-being		
	Development set	Validation set	*p-*Value^a^	Development set	Validation set	*p-*Value^a^	Development set	Validation set	*p-*Value^a^
	(*n* = 1320)	(*n* = 218)		(*n* =1247)	(*n* = 207)		(*n* =1319)	(*n* =219)	
Patient variables									
BMI^d^, mean (SD) (kg/m^2^)	26.63 (5.48)	25.72 (4.78)	**0.011**^**b**^	26.54 (5.43)	25.53 (4.76)	**0.006**^**b**^	26.61 (5.48)	25.70 (4.78)	**0.011**^**b**^
Preoperative patient-reported outcome data									
BREAST-Q physical well-being chest and upper body^d^, mean (SD), 0–100	78.46 (14.55)	81.22 (14.32)	**0.009**^**b**^	78.62 (14.37)	81.21 (14.09)	**0.015**^**b**^	78.45 (14.54)	81.24 (14.29)	**0.008**^**b**^
BREAST-Q physical well-being abdomen^d^, mean (SD), 0–100	89.26 (13.79)	91.07 (11.41)	**0.038**^**b**^	89.35 (13.55)	91.14 (11.48)	**0.046**^**b**^	89.27 (13.79)	91.03 (11.39)	**0.042**^**b**^
Clinical variables									
Reconstruction technique^d^									
Tissue expander (TE), no. (%)	691 (52.3)	129 (59.2)	0.061^c^	654 (52.4)	124 (59.9)	**0.046**^**c**^	690 (52.3)	130 (59.4)	0.053^c^
Superficial inferior epigastric artery (SIEA) flap, no. (%)	48 (3.6)	0 (0.00)	**0.004**^**c**^	42 (3.4)	0 (0.0)	**0.007**^**c**^	48 (3.6)	0 (0.0)	**0.004**^**c**^
Axillary intervention^d^									
Axillary lymph node dissection (ALND), no. (%)	354 (26.8)	43 (19.7)	**0.027**^**c**^	339 (27.2)	41 (19.8)	**0.025**^**c**^	352 (26.7)	43 (19.6)	**0.027**^**c**^
Sentinel lymph node biopsy (SLNB), no. (%)	579 (43.9)	112 (51.4)	**0.039**^**c**^	545 (43.7)	108 (52.2)	**0.023**^**c**^	579 (43.9)	113 (51.6)	**0.034**^**c**^
Socioeconomic and racial data									
Education level									
High school degree, no. (%)	113 (8.6)	9 (4.1)	**0.024**^**c**^	97 (7.8)	8 (3.9)	**0.043**^**c**^	112 (8.5)	9 (4.1)	**0.025**^**c**^
Masters/doctoral degree, no. (%)	382 (29.0)	88 (40.4)	**0.001**^**c**^	368 (29.6)	86 (41.5)	**0.001**^**c**^	387 (29.4)	88 (40.2)	**0.001**^**c**^
Working status									
Retired, no. (%)	128 (9.8)	11 (5.1)	**0.024**^**c**^	108 (8.8)	9 (4.4)	**0.033**^**c**^	129 (9.9)	11 (5.0)	**0.022**^**c**^
Part time employed, no. (%)	175 (13.4)	41 (18.9)	**0.033**^**c**^	164 (13.3)	40 (19.4)	**0.020**^**c**^	174 (13.3)	41 (18.8)	**0.032**^**c**^
Household income per year									
$25,000–49,999, no. (%)	147 (11.6)	14 (6.5)	**0.028**^**c**^	133 (11.0)	12 (5.9)	**0.025**^**c**^	146 (11.5)	14 (6.5)	**0.029**^**c**^
>$100,000, no. (%)	611 (48.1)	131 (61.2)	**0.0004**^**c**^	601 (49.9)	128 (62.7)	**0.001**^**c**^	617 (48.6)	132 (61.4)	**0.001**^**c**^
Outcome—patient-reported well-being at 2 year follow-up compared with baseline^d^									
Stable, no. (%)	199 (15.1)	39 (17.9)	0.287^c^	175 (14.0)	40 (19.3)	**0.047**^**c**^	265 (20.1)	51 (23.3)	0.278^c^

Table contains only significant comparison results between development and validation sets (see eTable 1 in Supplement 2 for more details). *p-*Values < 0.05 highlighted in bold.

^a^*p*-Values refer to differences in the development and validation set.

^b^*p*-Values refer to *t*-tests to evaluate mean differences of continuous data.

^c^*p-*Values refer to Chi-square tests for binary feature evaluation (feature true versus feature not true).

^d^ Variable included in the predictive models.

When comparing development and validation datasets, we observed significant differences in body mass index (BMI), baseline physical well-being, baseline physical well-being abdomen, superficial inferior epigastric artery (SIEA) flap, axillary lymph node dissection (ALND), sentinel lymph node biopsy (SLNB), high school degree, masters/doctoral degree, retired working status, part-time employed working status, $25,000–49,999 household income per year, and greater than $100,000 household income per year (all *p* < 0.05).

The correlation between 2 year psychosocial well-being and 2 year sexual well-being was highest (*r* = 0.72), followed by PRO scores at baseline (*r* = 0.63). The lowest level of correlation was observed between baseline physical well-being and 2 year sexual well-being (*r* = 0.14) (Table 2 in Supplement 1).

### Algorithm Performance

Table 2 displays the performance of ML models with tuned optimal hyperparameters (Table 3 in Supplement 1) in both test and validation sets for each subscale. In the validation set, AUC to predict worsened physical well-being was 0.69 (95%CI, 0.62–0.76) for the LR with elastic net penalty, 0.69 (95%CI, 0.62–0.76) for the XGBoost tree, and 0.70 (95%CI, 0.63–0.77) for the neural network. When predicting worsened sexual well-being, AUC of the three algorithms was 0.76 (95%CI, 0.70–0.82), 0.77 (95% CI, 0.70–0.83), and 0.77(95% CI, 0.70–0.83), respectively. When predicting worsened psychosocial well-being, AUCs were 0.66 (95% CI, 0.58–0.73), 0.66 (95% CI, 0.58–0.74), and 0.64 (95%CI, 0.55–0.72), respectively.

**Table 2:** Evaluation of algorithms trained to predict physical, sexual, psychosocial well-beings at 2 year follow-up

	2 Year follow-up score lower than baseline		2 Year follow-up score higher than baseline	
	Accuracy (95% CI)	AUC (95% CI)	Accuracy (95% CI)	AUC (95% CI)
Physical well-being				
Logistic regression with elastic net penalty				
Test set (*n* = 1320)	0.67(0.66–0.68)	0.71(0.70–0.72)	0.70(0.69–0.71)	0.77(0.75–0.78)
Additional validation set (*n* = 218)	0.63(0.56–0.69)	0.69(0.62–0.76)	0.76(0.69–0.81)	0.82(0.76–0.87)
XGBoost tree				
Test set (*n* = 1320)	0.66(0.64–0.67)	0.70(0.69–0.72)	0.70(0.69–0.71)	0.77(0.76–0.78)
Additional validation set (*n* = 218)	0.64(0.57–0.71)	0.69(0.62–0.76)	0.75(0.69–0.81)	0.81(0.75–0.86)
Neural network				
Test set (*n* = 1320)	0.65(0.64–0.67)	0.70(0.68–0.71)	0.69(0.69–0.71)	0.76(0.75–0.77)
Additional validation set (*n* = 218)	0.64 (0.58–0.71)	0.70(0.63–0.77)	0.75(0.69–0.81)	0.81(0.75–0.86)
Sexual well-being				
Logistic regression with elastic net penalty				
Test set (*n* = 1247)	0.69(0.68–0.70)	0.75(0.74–0.77)	0.72(0.71–0.74)	0.77(0.76–0.79)
Additional validation set (*n* = 207)	0.69(0.62–0.75)	0.76(0.70–0.82)	0.69(0.62–0.75)	0.76(0.69–0.82)
XGBoost tree				
Test set (*n* = 1247)	0.69(0.68–0.70)	0.75(0.74–0.76)	0.72(0.70–0.73)	0.76(0.74–0.77)
Additional validation set (*n* = 207)	0.70(0.63–0.76)	0.77(0.70–0.83)	0.69(0.62–0.75)	0.76(0.70–0.83)
Neural network				
Test set (*n* = 1247)	0.67(0.66–0.69)	0.75(0.73–0.76)	0.71(0.69–0.72)	0.77(0.76–0.79)
Additional validation set (*n* = 207)	0.70(0.63–0.76)	0.77(0.70–0.83)	0.67(0.60–0.73)	0.74(0.67–0.81)
Psychosocial well-being				
Logistic regression with elastic net penalty				
Test set (*n* = 1319)	0.73(0.71–0.74)	0.72(0.70–0.74)	0.69(0.68–0.70)	0.76(0.75–0.77)
Additional validation set (*n* = 219)	0.71(0.65–0.77)	0.66(0.58–0.73)	0.60(0.53–0.66)	0.66(0.59–0.73)
XGBoost tree				
Test set (*n* = 1319)	0.70(0.69–0.71)	0.68(0.66–0.70)	0.70(0.69–0.72)	0.77(0.76–0.79)
Additional validation set (*n* = 219)	0.70(0.63–0.76)	0.66(0.58–0.74)	0.62(0.55–0.68)	0.66(0.59–0.74)
Neural network				
Test set (*n* = 1319)	0.71(0.70–0.73)	0.72(0.70–0.73)	0.70(0.69–0.71)	0.76(0.75–0.78)
Additional validation set (*n* = 219)	0.71(0.64–0.77)	0.64(0.55–0.72)	0.60(0.53–0.66)	0.66(0.58–0.73)

*AUC* Area-under-the-receiver-operating-characteristic-curve

All three models were more proficient at predicting improved rather than worsened physical well-being, with AUCs of 0.82 (95% CI, 0.76–0.87), 0.81 (95% CI, 0.75–0.86), and 0.81 (95%CI, 0.75–0.86), respectively. In predicting improved sexual well-being, the AUC was 0.76, (95% CI, 0.69–0.82), 0.76 (95% CI, 0.70–0.83), and 0.74 (95% CI, 0.67–0.81), respectively. For improved psychosocial well-being, AUCs were 0.66 (95% CI, 0.59–0.73), 0.66 (95% CI, 0.59–0.74), and 0.66 (95% CI, 0.58–0.73).

**Fig. 2:**
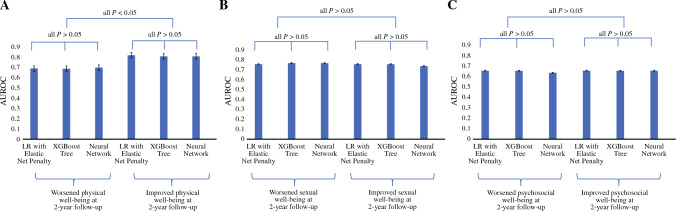
Performance comparison between machine learning (ml) models to predict improved and worsened physical, sexual, psychosocial well-beings with reconstructed breasts at 2 year follow-up. **A** ML models to predict physical well-being change. **B** ML models to predict sexual well-being change. **C** ML models to predict psychosocial well-being change

Figure 2 indicates that performance differences among the algorithms were not statistically significant (*p* > 0.05) except for the models predicting improved compared with worsened physical well-being (*p* < 0.05).

**Fig. 3:**
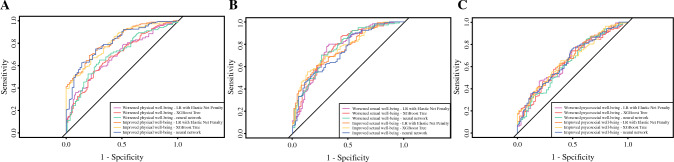
Receiver operating characteristic curves of machine learning models to predict improved and worsened physical, sexual, psychosocial well-beings with reconstructed breasts at 2 year follow-up. **A** Physical well-being with reconstructed breasts. **B** Sexual well-being with reconstructed breasts. **C** Psychosocial well-being with reconstructed breasts

An array of AUC curves of the models for each scale are displayed in Fig. 3. Calibration plots of all the models for each scale are presented in Figs. 1–3 of Supplement 3. Spiegelhalter’s Z test results (Table 4 Supplement 1) indicate that most of the models were well calibrated except for neural network in predicting worsened physical well-being (*p* = 0.01) and improved psychosocial well-being (*p* = 0.02), LR with elastic net penalty in predicting worsened sexual well-being (*p* = 0.003), and XGBoost tree in predicting worsened psychosocial well-being (*p* = 0.01) and improved psychosocial well-being (*p* = 0.0001).

### Predictive Coefficients and Variable Importance

The results (Table 3) indicate that preoperative physical well-being (*β*_regularized_, −1.08) was most strongly negatively correlated with improved physical well-being at 2 year follow-up.

**Table 3:** Regularized coefficients from the logistic regression with elastic net penalty

	Physical well-being (chest and upper body)		Sexual well-being		Psychosocial well-being	
	Regularized coefficient for worsened physical well-being at 2 year follow-up	Regularized coefficient for improved physical well-being at 2 year follow-up	Regularized coefficient for worsened sexual well-being at 2 year follow-up	Regularized coefficient for improved sexual well-being at 2 year follow-up	Regularized coefficient for worsened psychosocial well-being at 2 year follow-up	Regularized coefficient for improved psychosocial well-being at 2 year follow-up
Patient variables						
Age		− 0.03	− 0.06	0.15	− 0.18	0.06
BMI	0.01	− 0.10		− 0.01	0.09	
Diabetes						
Yes		− 0.14			− 0.38	0.23
No						
Smoker						
Never						
Previous		− 0.19		− 0.05	0.24	− 0.03
Current		− 0.14		− 0.12	0.07	− 0.03
Patient-reported outcomes at baseline						
Satisfaction with breasts		0.02	0.15	− 0.23	0.10	− 0.16
Psychosocial well-being		0.04	0.03	0.02	0.77	− 0.40
Physical well-being chest and upper body	0.43	− 1.08		0.02	− 0.05	− 0.01
Physical well-being abdomen		0.17			− 0.10	
Sexual well-being		− 0.07	0.32	− 0.78	− 0.08	− 0.08
Clinical variables						
Radiation						
After reconstruction	0.40	− 0.47	0.04	− 0.44	0.24	− 0.04
Before reconstruction		0.23	− 0.20	0.14	− 0.07	0.09
None		0.17			− 0.11	
Mastectomy						
Nipple sparing		− 0.02		0.14	− 0.25	
Simple						
Other		0.65			− 0.65	
Reconstruction						
Tissue expander (TE)		− 0.06	0.27	− 0.51	0.43	− 0.10
Direct-to-implant (DTI)		0.07			− 0.29	0.00
Transverse rectus abdominis (TRAM) flap		0.03	− 0.06	0.09	− 0.49	0.06
Deep inferior epigastric perforator (DIEP) flap	− 0.07	0.19	− 0.11	0.23	− 0.07	0.07
Latissimus dorsi (LD) flap		0.06		0.48	− 0.63	0.24
Gluteal artery perforator (GAP) flap		− 0.52			− 0.80	
Superficial inferior epigastric artery (SIEA) flap		− 0.15			− 1.04	0.10
Crossover flap		− 0.39		− 0.17	0.42	− 0.10
Mixed flaps		− 0.24		− 0.13	0.74	− 0.06
Mixed implants and autologous		0.36			− 0.88	0.12
Chemotherapy						
Received		0.06	0.15	− 0.21	0.26	− 0.19
Not received						
Laterality						
Unilateral reconstruction						
Bilateral reconstruction		− 0.01			0.07	
Mastectomy indication						
Therapeutic						
Prophylactic		0.17			− 0.36	
Axillary intervention						
Axillary lymph node dissection (ALND)	0.03	− 0.17	0.14	− 0.03	− 0.03	− 0.07
Sentinel lymph node biopsy (SLNB)		0.18			0.11	
None		− 0.04	− 0.11	0.05	− 0.08	0.04

Positive values indicate a positive correlation with the corresponding well-being, while negative values indicate a negative correlation with the corresponding well-being.

In predicting sexual well-being change at 2 year follow-up, baseline sexual well-being (*β*_regularized_, − 0.78), tissue expander (TE) reconstruction (*β*_regularized_, − 0.51), and radiation after reconstruction (*β*_regularized_, − 0.44), were most strongly negatively correlated with improved changes, whereas latissimus dorsi (LD) flap reconstruction (*β*_regularized_, 0.48) was positively correlated.

Additionally, superficial inferior epigastric artery (SIEA) flap reconstruction(*β*_regularized_, − 1.04), mixed implants and autologous reconstruction (*β*_regularized_, −0.88), GAP flap (*β*_regularized_, − 0.80), other types of mastectomy (*β*_regularized_, − 0.65), and latissimus dorsi (LD) flap reconstruction (*β*_regularized_, − 0.63) were most strongly negatively correlated with worsened psychosocial well-being at 2 year follow-up, whereas baseline psychosocial well-being (*β*_regularized_, 0.77) and mixed flaps reconstruction (β_regularized_, 0.74) were positively correlated with worsened outcomes. Similar variable importance, as well as its contribution to prediction, were also observed from XGBoost SHAP values and neural network LIME plots (Figs. 4 and 5 in Supplement 3), respectively.

The results of binary logistic regression identified key predictors and revealed their statistical significance in predicting changes in health-related QOL after surgery at 2 year follow-up (Table 5 in Supplement 1). When comparing the logistic regression with the coefficients of the ML models, generally the same direction and magnitude of associations could be observed with few exceptions. This gives credibility into the outcome predictions made by the ML model.

### Racial Bias Evaluation

The performance of all ML models in predicting both improved and worsened physical well-being statistically differed between the Caucasian and Asian groups (*p* < 0.05, higher scores for Asian subgroup). Neural networks performed statistically different between groups of Caucasian versus African American (*p* < 0.05, higher scores for African American subgroup), and African American versus Asian in predicting worsened sexual well-being (*p* < 0.05, higher scores for African American subgroup). All trained ML models showed statistically better performance for the African American group compared with the Caucasian group in both improved and worsened psychosocial well-being prediction (all *p* < 0.05) (Tables 6 and 7 in Supplement 1).

## Discussion

In this study, we developed and validated three ML algorithms to predict clinically meaningful, long-term changes in health-related QOL for women undergoing PMBR with acceptable accuracy. Our results indicate that baseline PRO data of physical, sexual, and psychosocial well-being had a much greater impact on long-term reported changes in QOL than clinical variables, revealing key predictors to consider when discussing expected QOL for patients undergoing cancer-related mastectomy.

We excluded study site as a variable in the machine learning model training for several reasons. First, neither the original study nor the present analysis considered the surgeon or study site as independent variables. Procedures were performed by 57 surgeons at 11 institutions, effectively balancing their influence despite potential skill variations. Second, adding study site as a variable would create a nonscalable algorithm that optimizes around potentially changing site characteristics. Therefore, excluding study site allows for better generalizability and scalability of the algorithm.

Compared with the sexual and psychosocial well-being of patients at 2 year follow-up, our findings show physical health improved in some patients but worsened in many more patients. This confirms previous findings that the physical well-being of the chest and upper body will not be fully restored, regardless of whether patients undergo implant-based or autologous reconstruction.^1^ The significant difference in performance between the prediction models for worsened and improved physical well-being suggests that the machine learning models encountered difficulties in accurately predicting worsened well-being. This could be attributed to (so far) unpredictable complications, such as infection, implant-related issues, or poor wound healing, which can arise during the postoperative period and result in suboptimal physical outcomes.

Previous studies using traditional statistical methods asserted that patients with autologous reconstruction tended to have a higher health-related QOL compared to those with implant-based reconstruction.^1,30^ Specifically, autologous reconstruction outperforms implants in tolerance of radiotherapy and improving QOL.^31^ Integrating radiotherapy with breast reconstruction results in a complex impact across multiple dimensions of a patients’ life.^31^ Patients with nipple-sparing mastectomy have significantly higher psychosocial and sexual well-being compared with patients with total mastectomy.^32^ However, inferences drawn from these group-level studies cannot infer specific treatment outcomes for individuals,^33^ as the relationships between variables of interest and outcomes are usually estimated after controlling for relevant co-variables, which does not reflect the real situation of each patient. Machine learning may overcome this limitation and help tailoring outcome predictions to the individual patient.^13^

Our results also indicate that patients with higher baseline physical, sexual, and psychosocial well-being were more likely to have worsened PROs in these three domains after breast reconstruction. Whether implant-based or autologous procedures were associated with improved or worsened QOL depended on the specific type of reconstruction, which was also seen in the binary logistic regression: taking direct-to-implant (DTI) reconstruction as a reference, TE reconstruction was associated with worsened sexual well-being and deep inferior epigastric perforator (DIEP) flap reconstruction was associated with a decreased risk of worsened physical well-being (chest and upper body). These individual-level outcomes predicted by ML models aim to better guide and optimize patient decision-making process to achieve expected postoperative outcomes when determining preferences for the exact reconstruction procedure. Nevertheless, a comparison of the performance of traditional statistical models with ML algorithms in this field appears highly warranted to help more clearly distinguish and highlight advantages of these developed intelligent decision-making tools.

Our team has previously published the development and validation of accurate ML algorithms to predict clinically meaningful changes in breast satisfaction with reconstructed breasts at 2 year follow-up in this cohort.^14^ AUC of the same three ML models to predict changes in satisfaction with reconstructed breasts study (improved: AUC range 0.86–0.87; decreased: AUC range 0.84–0.85) was higher compared with the performance in predicting changes in physical, sexual, and psychosocial well-being in the present analysis. Insights into predictors also underscored the importance of baseline PRO variables over clinical variables, similar to what we observed in the present analysis. Moreover, age was associated with worsened breast satisfaction (decreased: *β*_regularized_, 0.01) and physical well-being (improved: *β*_regularized_, − 0.03) but improved sexual well-being (improved: *β*_regularized_, 0.15) and psychosocial well-being (improved: *β*_regularized_, 0.06). This observation is underpinned by previous studies concluding that not all women necessarily experience worsening sexual function with higher age^34^ and that some older women have higher levels of sexual satisfaction,^35^ where psychosocial factors appear to play a crucial role.^36^

This study comes with several limitations. First, general guidelines for multivariate models recommend having at least 100 events for validation.^37^ However, none of the 11 study sites in this study met this requirement for all three scales simultaneously. Future, prospective validation with larger sample sizes seems warranted. Second, due to small samples or no samples in some racial groups (e.g., Hispanics), the ML performance assessment in these racial groups was not feasible. Some ML algorithms performed statistically significantly better in certain racial groups. We acknowledge that achieving equal performance across races becomes more challenging when sample sizes are limited, which aligns with findings of previous studies in this regard.^14^ Future studies may validate our findings and mitigate potential racial bias in a more diverse setting, not just academic institutions included here only. Third, although we achieved similar completion rates to similar PRO studies in literature,^1^ one cannot ignore that around half of the initially enrolled patients were lost to follow-up after 2 years. The results (Table 1 in Supplement 4) indicate that participants who were lost to follow-up were more likely to be younger, single, have a higher BMI, undergo TE reconstruction technique, and less likely to be married or have undergone DIEP reconstruction techniques. Future studies may consider using advanced PRO assessment approaches such as computer adaptive testing to reduce patients’ assessment burden and improve their engagement.^38,39^ Fourth, this study may establish a benchmark for interested researchers studying ML models on classification tasks for breast cancer patients. However, before implementing these algorithms in clinical practice to predict individual outcomes, necessary steps such as prospective clinical trials are needed to confirm their validity and reliability in real clinical settings.^33^ Fifth, the ML approach used in this study required dichotomization of outcomes. We chose this approach due to the recent upcoming concept of “clinically-important differences” in PROM research.^40,41^ However, some clinicians and patients might be more concerned about the degree or magnitude of change in their well-being due to the reconstruction procedure. This issue might be addressed in future research via modeling techniques that allow for continuous outcomes (e.g., linear modeling). Sixth, clinical implementation of digital health tools has proved to be a challenging task with many barriers including, for example, lack of transdisciplinary knowledge.^42^ Future research seems warranted to investigate the development and clinical feasibility of a digital tool that assists patients and clinicians in clinical decision-making process.

## Conclusions

In this study, we developed and validated ML algorithms to predict clinically meaningful, long-term changes in physical, sexual, and psychosocial well-being for women undergoing cancer-related mastectomy and breast reconstruction at 2 year follow-up with acceptable accuracy. These algorithms may function as a data-driven decision-making tool to assist in making informed treatment decisions for women undergoing breast reconstruction, and further facilitate patient-centered care by tailoring individualized treatment in clinical practice.

### Supplementary Information

Below is the link to the electronic supplementary material.

Supplementary file1 (DOCX 43 KB)

Supplementary file2 (DOCX 53 KB)

Supplementary file3 (DOCX 2235 KB)

Supplementary file4 (DOCX 48 KB)

## Data Availability

Individual participant data that underlie the results reported in this article, after deidentification (text, tables, figures, and appendices) be available (including data dictionaries) on reasonable request from the corresponding author.
