# Supplementary material for: Enhanced Surgical Decision-Making Tools in Breast Cancer: Predicting 2-Year Postoperative Physical, Sexual, and Psychosocial Well-Being following Mastectomy and Breast Reconstruction (INSPiRED 004)
Source: Ann Surg Oncol. 2023 Jul 30;30(12):7046–59. doi: 10.1245/s10434-023-13971-w (PMC10562277; doi:10.1245/s10434-023-13971-w)
Supplement: Supplementary file 1 — Supplementary file1 (DOCX 43 KB) [file 10434_2023_13971_MOESM1_ESM.docx]

**Supplement 1**

**Table 1. Candidate Variables for Algorithm Development**

| **Variable** | **Classification^a^** | **Brief description** |
| --- | --- | --- |
| Patient variables |  |  |
| Age | Numerical | Patient's age at the time of taking baseline Breast-Q assessments |
| BMI | Numerical | BMI as reported by the patient |
| Diabetes | Categorical | Diabetes if the patient had or not |
|  | (1)Yes |  |
|  | (2)No[reference] |  |
| Smoker | Either | Smoking status as reported by patients |
|  | (1)Previous |  |
|  | (2)Current |  |
|  | (3)Never[reference] |  |
| Pre-operative patient-reported outcome data |  |  |
| BREAST-Q satisfaction with breast | Numerical | Baseline score of breast satisfaction ranging from 0 to 100 reported by patients |
| BREAST-Q physical well-being chest and upper body | Numerical | Baseline score of physical well-being ranging from 0 to 100 reported by patients |
| BREAST-Q psychosocial well-being | Numerical | Baseline score of psychosocial well-being ranging from 0 to 100 reported by patients |
| BREAST-Q physical well-being abdomen | Numerical | Baseline score of physical well-being abdomen ranging from 0 to 100 reported by patients |
| BREAST-Q sexual well-being | Numerical | Baseline score of sexual well-being ranging from 0 to 100 reported by patients |
| Clinical variables |  |  |
| Radiation | Either | Radiation therapy received before or after reconstruction |
|  | (1)After reconstruction |  |
|  | (2)Before reconstruction |  |
|  | (3)None[reference] |  |
| Mastectomy | Either | Mastectomy type patients received |
|  | (1)Nipple-sparing |  |
|  | (2)Other |  |
|  | (3)Simple [Reference] |  |
| Reconstruction technique | Either | Reconstructive procedure type patients received |
|  | (1)Tissue expander (TE) |  |
|  | (2)Transverse rectus abdominis (TRAM) flap |  |
|  | (3)Deep inferior epigastric perforator (DIEP) flap |  |
|  | (4)Latissimus dorsi (LD) flap |  |
|  | (5)Gluteal artery perforator (GAP) flap |  |
|  | (6)Superficial inferior epigastric artery (SIEA) flap |  |
|  | (7)Crossover flap |  |
|  | (8)Mixed flaps |  |
|  | (9)Mixed implant and autologous |  |
|  | (10)Direct-to-implant (DTI)[reference] |  |
| Chemotherapy | Categorical | Chemotherapy received by patient during or after reconstruction |
|  | (1)Received |  |
|  | (2)Not received[reference] |  |
| Reconstruction laterality | Categorical | Reconstruction laterality type as reported by patients |
|  | (1)Unilateral |  |
|  | (2)Bilateral |  |
| Mastectomy indication | Categorical | Indication for mastectomy as reported by patients |
|  | (1)Prophylactic |  |
|  | (2)Therapeutic[reference] |  |
| Axillary intervention | Either | Axillary intervention type as reported by patients |
|  | (1)Axillary lymph node dissection (ALND) |  |
|  | (2)Sentinel lymph node biopsy (SLNB) |  |
|  | (3)None [Reference] |  |
| Socioeconomic and ethnic data |  |  |
| Marital status | Either | Marital status as reported by patients |
|  | (1)Single |  |
|  | (2)Living with significant other |  |
|  | (3)Married |  |
|  | (4)Separated |  |
|  | (5)Divorced |  |
|  | (6)Widowed |  |
|  |  |  |
| Education level | Either | Education level as reported by patients |
|  | (1)Some high school |  |
|  | (2)High school degree |  |
|  | (3)Some college/trade school |  |
|  | (4)College/trade school degree |  |
|  | (5)Some masters/doctoral |  |
|  | (6)Masters/doctoral degree |  |
| Working status | Either | Working status as reported by patients |
|  | (1)Unable to work |  |
|  | (2)Unemployed |  |
|  | (3)Student |  |
|  | (4)Volunteer |  |
|  | (5)Retired |  |
|  | (6)Homemaker |  |
|  | (7)Part time employed |  |
|  | (8)Full time employed |  |
|  | (9)Other |  |
| Household income per year | Either | Annual household income as reported by patients |
|  | (1)<25,000$ |  |
|  | (2)25,000$ to 49,999$ |  |
|  | (3)50,000$ to 74,999$ |  |
|  | (4)75,000$ to 99,999$ |  |
|  | (5)>100,000$ |  |
| Race background | Either | Race as reported by patients |
|  | (1)White |  |
|  | (2)Asian |  |
|  | (3)Black/African American |  |
|  | (4)American Indian/Alaska Native |  |
|  | (5)Native Hawaiian/Other Pacific Islander |  |
| Outcome—patient-reported physical well-being at 2-year follow-up compared to  baseline | Either | The changes of physical well-being equal or greater than minimal clinically important difference of 3 defines the 3 outcome types of improved, decreased, unchanged physical well-being for patients |
|  | (1)Improved |  |
|  | (2)Worsened |  |
|  | (3)Stable |  |

^a^It is methodologically necessary to dichotomize categorical variables.

|  | **Baseline**  **PRO.physical** | **Baseline**  **PRO.psychosocial** | **Baseline**  **PRO.sexual** | **2year**  **PRO.physical** | **2year**  **PRO.psychosocial** | **2year**  **PRO.sexual** |
| --- | --- | --- | --- | --- | --- | --- |
| BaselinePRO.physical | 1 |  |  |  |  |  |
| BaselinePRO.psychosocial | 0.31 | 1 |  |  |  |  |
| BaselinePRO.sexual | 0.23 | 0.63 | 1 |  |  |  |
| 2yearPRO.physical | 0.44 | 0.21 | 0.15 | 1 |  |  |
| 2yearPRO.psychosocial | 0.20 | 0.38 | 0.27 | 0.40 | 1 |  |
| 2yearPRO.sexual | 0.14 | 0.29 | 0.36 | 0.34 | 0.72 | 1 |

**Table 2. Pearson-correlation among Physical, Sexual, and Psychosocial Scales at Baseline and 2-Year Follow-up**

**Table 3. Best Hyperparameters Selected for Machine Learning Algorithms to Predict Physical, Sexual, Psychosocial Well-beings with Reconstructed Breasts**

| **Models^a^** | **Hyperparameter** | **Worsened physical**  **well-being** | | **Improved physical**  **well-being** | | **Worsened sexual**  **well-being** | | **Improved sexual**  **well-being** | | **Worsened psychosocial**  **well-being** | | **Improved psychosocial**  **well-being** | |
| --- | --- | --- | --- | --- | --- | --- | --- | --- | --- | --- | --- | --- | --- |
|  |  | Optimal hyperparameters | Kappa value,  (Sd) | Optimal  hyperparameters | Kappa value,  (Sd) | Optimal hyperparameters | Kappa value, (Sd) | Optimal hyperparameters | Kappa value, (Sd) | Optimal hyperparameters | Kappa value,  (Sd) | Optimal hyperparameters | Kappa value,  (Sd) |
| Logistic regression with elastic net penalty |  |  | 0.33  (0.07) |  | 0.33  (0.08) |  | 0.36  (0.07) |  | 0.41  (0.08) |  | 0.24  (0.08) |  | 0.38  (0.07) |
|  | alpha | 0.23 |  | 0.01 |  | 0.1 |  | 0.38 |  | 0.01 |  | 0.04 |  |
|  | lambda | 0.11 |  | 0.02 |  | 0.21 |  | 0.02 |  | 0.01 |  | 0.23 |  |
| XGBoost tree |  |  | 0.31  (0.08) |  | 0.35  (0.08) |  | 0.37  (0.07) |  | 0.40  (0.09) |  | 0.20  (0.07) |  | 0.40  (0.08) |
|  | nrounds | 214 |  | 532 |  | 627 |  | 958 |  | 367 |  | 197 |  |
|  | max_depth | 1 |  | 2 |  | 1 |  | 4 |  | 4 |  | 2 |  |
|  | eta | 0.07 |  | 0.04 |  | 0.36 |  | 0.11 |  | 0.51 |  | 0.09 |  |
|  | gamma | 8.91 |  | 9.78 |  | 5.40 |  | 7.47 |  | 7.48 |  | 3.06 |  |
|  | colsample_bytree | 0.63 |  | 0.36 |  | 0.64 |  | 0.56 |  | 0.65 |  | 0.42 |  |
|  | min_child_weight | 5 |  | 17 |  | 9 |  | 13 |  | 12 |  | 8 |  |
|  | subsample | 0.72 |  | 0.59 |  | 0.83 |  | 0.36 |  | 0.56 |  | 0.27 |  |
| Neural network |  |  | 0.30  (0.08) |  | 0.32  (0.08) |  | 0.34  (0.08) |  | 0.39  (0.09) |  | 0.23  (0.09) |  | 0.40  (0.07) |
|  | size | 11 |  | 10 |  | 10 |  | 11 |  | 11 |  | 11 |  |
|  | dropout | 0.01 |  | 0.19 |  | 0.19 |  | 0.01 |  | 0.01 |  | 0.01 |  |
|  | batch_size | 495 |  | 839 |  | 793 |  | 467 |  | 494 |  | 494 |  |
|  | lr | 0.36 |  | 0.30 |  | 0.30 |  | 0.36 |  | 0.36 |  | 0.36 |  |
|  | rho | 0.29 |  | 0.02 |  | 0.02 |  | 0.29 |  | 0.29 |  | 0.29 |  |
|  | decay | 0.13 |  | 0.43 |  | 0.43 |  | 0.13 |  | 0.13 |  | 0.13 |  |
|  | cost | 6.01 |  | 2.53 |  | 2.53 |  | 6.01 |  | 6.01 |  | 6.01 |  |

Note: 10-fold cross-validation and the hypergrid were used to tune hyperparameters for logistic regression with elastic net penalty, XGBoost tree (random grid search), and neural network (random grid search).

**Table 4. Spiegelhalter’s Z test for Machine Learning Algorithms Calibration Evaluation in Validation Set**

|  | **Z-score** | ***P* value^a^** | **Conclusion** | **Brier-score^b^** |
| --- | --- | --- | --- | --- |
| **Worsened physical well-being** |  |  |  |  |
| Logistic regression with elastic net penalty | -1.92 | **0.03** | Not well calibrated | 0.22 |
| XGBoost tree | -0.39 | 0.35 | Well calibrated | 0.22 |
| Neural network | 2.61 | **0.01** | Not well calibrated | 0.23 |
| **Improved physical well-being** |  |  |  |  |
| Logistic regression with elastic net penalty | -1.69 | 0.05 | Well calibrated | 0.16 |
| XGBoost tree | -1.57 | 0.06 | Well calibrated | 0.17 |
| Neural network | -0.45 | 0.33 | Well calibrated | 0.16 |
| **Worsened sexual well-being** |  |  |  |  |
| Logistic regression with elastic net penalty | -2.76 | **<0.001** | Not well calibrated | 0.20 |
| XGBoost tree | -0.30 | 0.38 | Well calibrated | 0.19 |
| Neural network | 1.49 | 0.07 | Well calibrated | 0.20 |
| **Improved sexual well-being** |  |  |  |  |
| Logistic regression with elastic net penalty | -0.30 | 0.38 | Well calibrated | 0.19 |
| XGBoost tree | -0.10 | 0.46 | Well calibrated | 0.19 |
| Neural network | 1.30 | 0.10 | Well calibrated | 0.20 |
| **Worsened psychosocial well-being** |  |  |  |  |
| Logistic regression with elastic net penalty | 0.36 | 0.36 | Well calibrated | 0.19 |
| XGBoost tree | 2.20 | **0.01** | Not well calibrated | 0.20 |
| Neural network | 0.55 | 0.29 | Well calibrated | 0.20 |
| **Improved psychosocial well-being** |  |  |  |  |
| Logistic regression with elastic net penalty | -0.24 | 0.40 | Well calibrated | 0.23 |
| XGBoost tree | 3.71 | **<0.001** | Not well calibrated | 0.24 |
| Neural network | 1.96 | **0.02** | Not well calibrated | 0.23 |

^a^*P* values < 0.05 highlighted in bold.

^b^Brier score with range of 0 to 1 denotes the calibration level of perfect to poor.

**Table 5. Multivariable Logistic Regression for Health-related Quality of Life with Reconstructed Breasts**

|  | **Physical well-being (chest and upper body)** | |  | **Sexual well-being** | |  | **Psychosocial well-being** | |
| --- | --- | --- | --- | --- | --- | --- | --- | --- |
|  | Worsened physical  well-being | Improved physical  well-being |  | Worsened sexual  well-being | Improved sexual  well-being |  | Worsened psychosocial well-being | Improved psychosocial well-being |
|  | Odds ratio  (95% CI) | Odds ratio  (95% CI) |  | Odds ratio  (95% CI) | Odds ratio  (95% CI) |  | Odds ratio  (95% CI) | Odds ratio  (95% CI) |
| **Patient variables** |  |  |  |  |  |  |  |  |
| Age | 1.00  (0.98-1.01) | 1.00  (0.98-1.01) |  | 0.99  (0.98-1.00) | 1.02*  (1.00-1.03) |  | 0.98**  (0.97-0.99) | 1.02**  (1.01-1.04) |
| BMI | 1.03**  (1.01-1.06) | 0.97*  (0.94-1.00) |  | 1.01  (0.98-1.04) | 0.98  (0.95-1.01) |  | 1.02  (1.00 -1.05) | 0.99  (0.96-1.01) |
| Diabetes |  |  |  |  |  |  |  |  |
| Yes | 1.11  (0.63-1.95) | 0.99  (0.52-1.84) |  | 1.39  (0.73-2.62) | 1.09  (0.58-2.04) |  | 0.75  (0.36-1.47) | 2.09*  (1.11-4.10) |
| No | 1 [reference] | 1 [reference] |  | 1[reference] | 1[reference] |  | 1[reference] | 1[reference] |
| Smoker |  |  |  |  |  |  |  |  |
| Previous | 1.28*  (1.00-1.64) | 0.77  (0.59-1.02) |  | 1.16  (0.89-1.51) | 0.86  (0.65-1.13) |  | 1.11  (0.85-1.46) | 0.84  (0.64-1.09) |
| Current | 1.16  (0.47-2.86) | 0.85  (0.32-2.21) |  | 0.70  (0.25-1.82) | 0.59  (0.21-1.57) |  | 0.86  (0.28-2.37) | 0.66  (0.25-1.72) |
| Never | 1 [reference] | 1 [reference] |  | 1[reference] | 1[reference] |  | 1[reference] | 1[reference] |
| **Patient-reported outcomes at baseline** |  |  |  |  |  |  |  |  |
| Satisfaction with breasts | 1.00  (0.99-1.01) | 1.00  (0.99-1.01) |  | 1.01***  (1.01-1.02) | 0.99***  (0.98-0.99) |  | 1.00  (1.00-1.01) | 0.99  (0.99-1.00) |
| Psychosocial well-being | 1.00  (0.99-1.00) | 1.01  (1.00-1.02) |  | 0.99*  (0.98-1.00) | 1.01**  (1.00-1.02) |  | 1.05***  (1.04-1.06) | 0.94***  (0.93-0.95) |
| Physical well-being chest and upper body | 1.06***  (1.05-1.07) | 0.91***  (0.90-0.92) |  | 0.99*  (0.98-1.00) | 1.00  (0.99-1.01) |  | 0.99  (0.98-1.00) | 1.00  (0.99-1.01) |
| Physical well-being abdomen | 0.98***  (0.97-0.99) | 1.02***  (1.01-1.03) |  | 1.00  (0.99-1.01) | 1.00  (0.99-1.01) |  | 0.99  (0.98-1.00) | 1.00  (0.99-1.01) |
| Sexual well-being | 1.00  (0.99-1.01) | 1.00  (0.99-1.00) |  | 1.05***  (1.04-1.06) | 0.95***  (0.94-0.96) |  | 0.99  (0.99-1.00) | 1.01*  (1.00-1.02) |
| **Clinical variables** |  |  |  |  |  |  |  |  |
| Radiation |  |  |  |  |  |  |  |  |
| After reconstruction | 2.53***  (1.77-3.65) | 0.42***  (0.27-0.63) |  | 1.23  (0.85-1.78) | 0.60*  (0.40-0.89) |  | 1.68**  (1.16-2.44) | 0.77  (0.53-1.12) |
| Before reconstruction | 1.01  (0.69-1.48) | 1.05  (0.70-1.59) |  | 0.84  (0.53-1.31) | 0.98  (0.64-1.49) |  | 1.02  (0.64-1.60) | 1.21  (0.81-1.83) |
| None | 1[reference] | 1[reference] |  | 1[reference] | 1[reference] |  | 1[reference] | 1[reference] |
| Mastectomy |  |  |  |  |  |  |  |  |
| Nipple-sparing | 1.17  (0.80-1.72) | 1.08  (0.70-1.65) |  | 0.80  (0.54-1.20) | 1.41  (0.92-2.15) |  | 0.83  (0.54-1.25) | 0.93  (0.62-1.40) |
| Other | 0.74  (0.10-4.01) | 2.64  (0.47-20.29) |  | 0.33  (0.04-1.95) | 1.52  (0.19-8.91) |  | 0.38  (0.02-2.71) | 0.95  (0.15-6.10) |
| Simple | 1[reference] | 1[reference] |  | 1[reference] | 1[reference] |  | 1[reference] | 1[reference] |
| Reconstruction |  |  |  |  |  |  |  |  |
| TE | 0.84  (0.49-1.45) | 0.94  (0.52-1.75) |  | 1.99*  (1.13-3.55) | 0.53*  (0.30-0.96) |  | 1.80  (0.98-3.47) | 0.93  (0.53-1.65) |
| TRAM | 0.60  (0.30-1.18) | 0.98  (0.46-2.11) |  | 1.19  (0.57-2.50) | 1.08  (0.53-2.24) |  | 0.83  (0.37-1.90) | 1.28  (0.62-2.64) |
| DIEP | 0.52*  (0.28-0.95) | 1.21  (0.63-2.36) |  | 0.84  (0.45-1.59) | 1.34  (0.72-2.54) |  | 1.09  (0.56-2.23) | 1.47  (0.79-2.75) |
| LD | 0.71  (0.30-1.64) | 0.90  (0.35-2.28) |  | 0.85  (0.32-2.19) | 1.86  (0.73-4.85) |  | 0.68  (0.22-1.94) | 2.14  (0.86-5.48) |
| GAP | 1.34  (0.26-7.98) | 0.49  (0.05-3.23) |  | 0.55  (0.07-3.13) | 1.16  (0.22-6.38) |  | 0.48  (0.02-3.31) | 0.54  (0.09-2.80) |
| SIEA | 0.58  (0.24-1.37) | 0.77  (0.29-2.02) |  | 0.85  (0.33-2.11) | 1.09  (0.44-2.70) |  | 0.39  (0.11-1.20) | 1.66  (0.68 -4.12) |
| Crossover | 0.76  (0.34-1.69) | 0.67  (0.27-1.66) |  | 1.75  (0.77-3.99) | 0.72  (0.31-1.68) |  | 1.57  (0.66-3.76) | 1.02  (0.45-2.33) |
| Mixed flaps | 0.68  (0.29-1.58) | 0.95  (0.37-2.42) |  | 1.49  (0.60-3.66) | 0.83  (0.34-2.03) |  | 2.14  (0.84-5.50) | 0.74  (0.31-1.80) |
| Mixed implants and autologous | 0.77  (0.27-2.11) | 1.40  (0.48-4.21) |  | 0.98  (0.26-3.27) | 1.14  (0.38-3.57) |  | 0.48  (0.09-1.88) | 1.95  (0.62-6.45) |
| DTI | 1[reference] | 1[reference] |  | 1[reference] | 1[reference] |  | 1[reference] | 1[reference] |
| Chemotherapy |  |  |  |  |  |  |  |  |
| Yes | 1.03  (0.78-1.37) | 1.11  (0.81-1.51) |  | 1.50**  (1.11-2.01) | 0.76  (0.56-1.05) |  | 1.17  (0.87-1.58) | 0.68**  (0.50-0.91) |
| No | 1[reference] | 1[reference] |  | 1[reference] | 1[reference] |  | 1[reference] | 1[reference] |
| Laterality |  |  |  |  |  |  |  |  |
| Bilateral reconstruction | 0.93  (0.73-1.20) | 0.99  (0.75-1.31) |  | 1.12  (0.86-1.46) | 0.89  (0.68-1.17) |  | 1.13  (0.86-1.48) | 1.07  (0.82-1.38) |
| Unilateral reconstruction | 1[reference] | 1[reference] |  | 1[reference] | 1[reference] |  | 1[reference] | 1[reference] |
| Mastectomy indication |  |  |  |  |  |  |  |  |
| Prophylactic indication | 0.77  (0.49-1.20) | 1.29  (0.78-2.13) |  | 1.72*  (1.05-2.82) | 0.88  (0.54-1.44) |  | 0.67  (0.39-1.14) | 0.97  (0.61-1.55) |
| Therapeutic indication | 1[reference] | 1[reference] |  | 1[reference] | 1[reference] |  | 1[reference] | 1[reference] |
| Axillary intervention |  |  |  |  |  |  |  |  |
| ALND | 0.96  (0.65-1.42) | 1.02  (0.66-1.57) |  | 1.76**  (1.15-2.69) | 0.83  (0.54-1.28) |  | 1.23  (0.80-1.90) | 0.86  (0.57-1.30) |
| SLNB | 0.80  (0.58-1.10) | 1.43*  (1.01-2.03) |  | 1.28  (0.90-1.83) | 0.90  (0.64-1.27) |  | 1.35  (0.94-1.94) | 0.98  (0.70-1.36) |
| None | 1[reference] | 1[reference] |  | 1[reference] | 1[reference] |  | 1[reference] | 1[reference] |

Note: **p*<0.05; ***p*<0.01; ****p*<0.001.

**Table 6. Performance of Machine Learning Models by Race for Quality of Life Change with Reconstructed Breast in Validation Dataset**

|  | N | Logistic regression with elastic net penalty |  | XGBoost tree |  | Neural network |
| --- | --- | --- | --- | --- | --- | --- |
|  |  | AUC(95%CI) |  | AUC(95%CI) |  | AUC(95%CI) |
| Worsened physical well-being |  |  |  |  |  |  |
| Caucasian | 198 | 0.68(0.60-0.75) |  | 0.68(0.61-0.75) |  | 0.70(0.62-0.77) |
| Asian | 7 | 0.92(0.67-1) |  | 0.92(0.67-1) |  | 1(1-1) |
| African American | 8 | 0.67 (0.17-1) |  | 0.83(0.5-1) |  | 0.5(0-1) |
| American Indian/Alaska Native | 1 | NA |  | NA |  | NA |
| Native Hawaiian or Other Pacific Islander | 1 | NA |  | NA |  | NA |
| Improved physical well-being |  |  |  |  |  |  |
| Caucasian | 198 | 0.82(0.76-0.87) |  | 0.80(0.75-0.86) |  | 0.81(0.74-0.87) |
| Asian | 7 | 1(1-1) |  | 1(1-1) |  | 1(1-1) |
| African American | 8 | 0.67(0.17-1) |  | 0.83(0.33-1) |  | 0.58(0-1) |
| American Indian/Alaska Native | 1 | NA |  | NA |  | NA |
| Native Hawaiian or Other Pacific Islander | 1 | NA |  | NA |  | NA |
| Worsened sexual well-being |  |  |  |  |  |  |
| Caucasian | 188 | 0.76(0.69-0.83) |  | 0.77(0.70-0.84) |  | 0.77(0.70-0.84) |
| Asian | 7 | 0.5(0-1) |  | 0.5(0-1) |  | 0.42(0-1) |
| African American | 7 | 0.9(0.6-1) |  | 0.9(0.6-1) |  | 1(1-1) |
| American Indian/Alaska Native | 1 | NA |  | NA |  | NA |
| Native Hawaiian or Other Pacific Islander | 1 | NA |  | NA |  | NA |
| Improved sexual well-being |  |  |  |  |  |  |
| Caucasian | 188 | 0.75(0.67-0.81) |  | 0.76(0.68-0.82) |  | 0.73(0.65-0.80) |
| Asian | 7 | 0.67(0.33-1) |  | 0.67(0.33-1) |  | 0.83(0.5-1) |
| African American | 7 | 0.92(0.66-1) |  | 0.92(0.67-1) |  | 0.92(0.67-1) |
| American Indian/Alaska Native | 1 | NA |  | NA |  | NA |
| Native Hawaiian or Other Pacific Islander | 1 | NA |  | NA |  | NA |
| Worsened psychosocial well-being |  |  |  |  |  |  |
| Caucasian | 198 | 0.66(0.57-0.74) |  | 0.66(0.57-0.73) |  | 0.64(0.56-0.73) |
| Asian | 8 | NA |  | NA |  | NA |
| African American | 8 | 1(1-1) |  | 1(1-1) |  | 1(1-1) |
| American Indian/Alaska Native | 1 | NA |  | NA |  | NA |
| Native Hawaiian or Other Pacific Islander | 1 | NA |  | NA |  | NA |
| Improved psychosocial well-being |  |  |  |  |  |  |
| Caucasian | 198 | 0.66(0.59-0.73) |  | 0.67(0.59-0.74) |  | 0.66(0.58-0.73) |
| Asian | 8 | 0.58(0.17-1) |  | 0.5(0.17-0.83) |  | 0.5(0.17-0.83) |
| African American | 8 | 0.92(0.67-1) |  | 1(1-1) |  | 0.92(0.67-1) |
| American Indian/Alaska Native | 1 | NA |  | NA |  | NA |
| Native Hawaiian or Other Pacific Islander | 1 | NA |  | NA |  | NA |

Note: NA denotes AUC curve was not able to be plotted due to smaller number of patients or one level left in outcome variables.

**Table 7. Performance Comparison of Machine Learning Models by Race for Quality of Life Change with Reconstructed Breast in Validation Dataset**

|  | | | _Worsened physical well-being_ |  | _Improved physical well-being_ |  | _Worsened sexual well-being_ |  | _Improved sexual well-being_ |  | _Worsened psychosocial well-being_ |  | _Improved psychosocial well-being_ |
| --- | --- | --- | --- | --- | --- | --- | --- | --- | --- | --- | --- | --- | --- |
|  | | | *_P_*_-value_ |  | *_P_*_-value_ |  | *_P_*_-value_ |  | *_P_*_-value_ |  | *_P_*_-value_ |  | *_P_*_-value_ |
| _Logistic regression with elastic net penalty_ | | |  |  |  |  |  |  |  |  |  |  |  |
| _Caucasian_ | _vs_ | _African American_ | 0.96 |  | 0.57 |  | 0.32 |  | 0.16 |  | <0.001 |  | 0.03 |
| _Caucasian_ | _vs_ | _Asian_ | 0.05 |  | <0.001 |  | 0.32 |  | 0.68 |  | NA |  | 0.70 |
| _African American_ | _vs_ | _Asian_ | 0.38 |  | 0.20 |  | 0.17 |  | 0.26 |  | NA |  | 0.14 |
| _XGBoost tree_ |  |  |  |  |  |  |  |  |  |  |  |  |  |
| _Caucasian_ | _vs_ | _African American_ | 0.37 |  | 0.86 |  | 0.33 |  | 0.19 |  | <0.001 |  | <0.001 |
| _Caucasian_ | _vs_ | _Asian_ | 0.04 |  | <0.001 |  | 0.30 |  | 0.64 |  | NA |  | 0.45 |
| _African American_ | _vs_ | _Asian_ | 0.67 |  | 0.32 |  | 0.16 |  | 0.26 |  | NA |  | 0.02 |
| _Neural network_ |  |  |  |  |  |  |  |  |  |  |  |  |  |
| _Caucasian_ | _vs_ | _African American_ | 0.59 |  | 0.47 |  | <0.001 |  | 0.12 |  | <0.001 |  | 0.03 |
| _Caucasian_ | _vs_ | _Asian_ | <0.001 |  | <0.001 |  | 0.14 |  | 0.51 |  | NA |  | 0.45 |
| _African American_ | _vs_ | _Asian_ | 0.16 |  | 0.17 |  | 0.01 |  | 0.66 |  | NA |  | 0.08 |

Note: *P* values < 0.05 highlighted in bold.
