# Supplementary material for: Enhanced Surgical Decision-Making Tools in Breast Cancer: Predicting 2-Year Postoperative Physical, Sexual, and Psychosocial Well-Being following Mastectomy and Breast Reconstruction (INSPiRED 004)
Source: Ann Surg Oncol. 2023 Jul 30;30(12):7046–59. doi: 10.1245/s10434-023-13971-w (PMC10562277; doi:10.1245/s10434-023-13971-w)
Supplement: Supplementary file 2 — Supplementary file2 (DOCX 53 KB) [file 10434_2023_13971_MOESM2_ESM.docx]

**Supplement 2**

**Table 1**. **Participant Baseline Characteristics and Health Outcomes**

|  | **Physical well-being (chest and upper body)** | | | | **Sexual well-being** | | | | **Psychosocial well-being** | | | |
| --- | --- | --- | --- | --- | --- | --- | --- | --- | --- | --- | --- | --- |
|  | Whole cohort | Development set | Validation set | *P* value^a^ | Whole cohort | Development set | Validation set | *P* value^a^ | Whole cohort | Development set | Validation set | *P* value^a^ |
|  | (n=1538) | (n= 1320) | (n= 218) |  | (n=1454) | (n=1247) | (n= 207) |  | (n=1538) | (n=1319) | (n=219) |  |
| **Patient variables** |  |  |  |  |  |  |  |  |  |  |  |  |
| Age^e^, mean (SD), years | 50.25(10.00) | 50.29(10.12) | 50.03(9.21) | 0.704^b^ | 49.86(9.87) | 49.87(9.99) | 49.80(9.12) | 0.921^b^ | 50.19(9.97) | 50.22(10.09) | 49.99(9.21) | 0.732^b^ |
| BMI^e^, mean (SD), kg/m^2^ | 26.50(5.40) | 26.63(5.48) | 25.72(4.78) | **0.011^b^** | 26.40(5.35) | 26.54(5.43) | 25.53(4.76) | **0.006^b^** | 26.48(5.39) | 26.61(5.48) | 25.70(4.78) | **0.011^b^** |
| Diabetes^e^, no (%) |  |  |  | 0.472^c^ |  |  |  | 0.274^c^ |  |  |  | 0.463^c^ |
| No, no. (%) | 1467 (95.4) | 1257(95.2) | 210(96.3) |  | 1391(95.7) | 1190(95.4) | 201(97.1) |  | 1467(95.4) | 1256(95.2) | 211(96.3) |  |
| Yes, no. (%) | 71(4.6) | 63(4.8) | 8(3.7) |  | 63(4.3) | 57(4.6) | 6(2.9) |  | 71(4.6) | 63(4.8) | 8(3.7) |  |
| Smoker^e^ |  |  |  |  |  |  |  |  |  |  |  |  |
| Never, no. (%) | 1015(66.6) | 874(66.8) | 141(65.3) | 0.667^c^ | 971(67.3) | 837(67.7) | 134(65.0) | 0.449^c^ | 1020(66.9) | 878(67.1) | 142(65.4) | 0.625^c^ |
| Previous, no. (%) | 483(31.7) | 410(31.3) | 73(33.8) | 0.469^c^ | 446(30.9) | 376(30.4) | 70(34.0) | 0.306^c^ | 479(31.4) | 406(31.0) | 73(33.6) | 0.445^c^ |
| Current, no. (%) | 27(1.8) | 25(1.9) | 2(0.9) | 0.461^c^ | 25(1.7) | 23(1.9) | 2(1.0) | 0.537^c^ | 26(1.7) | 24(1.8) | 2(0.9) | 0.497^c^ |
| Unknown, no. (%) | 13(0.8) | 11(0.8) | 2(0.9) | 1^c^ | 12(0.8) | 11(0.9) | 1(0.5) | 0.863^c^ | 13(0.8) | 11(0.8) | 2(0.9) | 1^c^ |
| **Pre-operative patient-reported outcome data** |  |  |  |  |  |  |  |  |  |  |  |  |
| BREAST-Q satisfaction with breast^e^, mean (SD), 0-100 | 60.06(22.17) | 59.84(22.15) | 61.38(22.28) | 0.346^b^ | 60.25(22.20) | 59.990(22.15) | 61.81(22.47) | 0.281^b^ | 60.07(22.13) | 59.84(22.11) | 61.46(22.26) | 0.319^b^ |
| BREAST-Q physical well-being chest and upper body^e^, mean (SD), 0-100 | 78.85(14.54) | 78.46(14.55) | 81.22(14.32) | **0.009^b^** | 78.99(14.36) | 78.62 (14.37) | 81.21(14.09) | **0.015^b^** | 78.85(14.54) | 78.45(14.54) | 81.24(14.29) | **0.008^b^** |
| BREAST-Q psychosocial well-being^e^, mean (SD), 0-100 | 69.64(18.13) | 69.58(18.18) | 70.01(17.89) | 0.738^b^ | 69.53(18.09) | 69.46(18.10) | 69.96(18.09) | 0.714^b^ | 69.62(18.09) | 69.54(18.13) | 70.07(17.86) | 0.686^b^ |
| BREAST-Q physical well-being abdomen^e^, mean (SD), 0-100 | 89.51(13.49) | 89.26(13.79) | 91.07(11.41) | **0.038^b^** | 89.60(13.29) | 89.35(13.55) | 91.14(11.48) | **0.046^b^** | 89.51(13.49) | 89.27(13.79) | 91.03(11.39) | **0.042^b^** |
| BREAST-Q sexual well-being^e^, mean (SD), 0-100 | 54.85(20.74) | 55.00(20.62) | 53.88(21.46) | 0.477^b^ | 55.37(20.44) | 55.52(20.26) | 54.46(21.50) | 0.507^b^ | 54.94(20.72) | 55.08(20.57) | 54.09(21.64) | 0.538^b^ |
| **Clinical variables** |  |  |  |  |  |  |  |  |  |  |  |  |
| Radiation^e^ |  |  |  |  |  |  |  |  |  |  |  |  |
| After reconstruction, no. (%) | 287(18.7) | 247(18.7) | 40(18.3) | 0.898^c^ | 274(18.8) | 235(18.8) | 39(18.8) | 0.999^c^ | 287(18.7) | 247(18.7) | 40(18.3) | 0.871^c^ |
| Before reconstruction, no. (%) | 224(14.6) | 190(14.4) | 34(15.6) | 0.641^c^ | 201(13.8) | 170(13.6) | 31(15.0) | 0.604^c^ | 220(14.3) | 186(14.1) | 34(15.5) | 0.577^c^ |
| None, no. (%) | 1027(66.8) | 883(66.9) | 144(66.1) | 0.808^c^ | 979(67.3) | 842(67.5) | 137(66.2) | 0.704^c^ | 1031(67.0) | 886(67.2) | 145(66.2) | 0.779^c^ |
| Mastectomy^e^ |  |  |  |  |  |  |  |  |  |  |  |  |
| Nipple-sparing, no. (%) | 166(10.8) | 145(11.0) | 21(9.6) | 0.551^c^ | 162(11.1) | 143(11.5) | 19(9.2) | 0.332^c^ | 168(10.9) | 147(11.1) | 21(9.6) | 0.494^c^ |
| Simple, no. (%) | 1366(88.8) | 1170(88.6) | 196(89.9) | 0.581^c^ | 1286(88.4) | 1099(88.1) | 187(90.3) | 0.358^c^ | 1364(88.7) | 1167(88.5) | 197(90.0) | 0.522^c^ |
| Other, no. (%) | 6(0.4) | 5(0.4) | 1(0.5) | 1^c^ | 6(0.4) | 5(0.4) | 1(0.5) | 1^c^ | 6(0.4) | 5(0.4) | 1(0.5) | 1^c^ |
| Reconstruction technique^e^ |  |  |  |  |  |  |  |  |  |  |  |  |
| Tissue expander (TE), no. (%) | 820(53.3) | 691(52.3) | 129(59.2) | 0.061^c^ | 778(53.5) | 654(52.4) | 124(59.9) | **0.046^c^** | 820(53.3) | 690(52.3) | 130(59.4) | 0.053^c^ |
| Direct-to-implant (DTI), no. (%) | 71(4.6) | 62(4.7) | 9(4.1) | 0.711^c^ | 67(4.6) | 59(4.7) | 8(3.9) | 0.582^c^ | 71(4.6) | 62(4.7) | 9(4.1) | 0.700^c^ |
| Transverse rectus abdominis (TRAM) flap, no. (%) | 120(7.8) | 99(7.5) | 21(9.6) | 0.277^c^ | 115(7.9) | 94(7.5) | 21(10.1) | 0.198^c^ | 120(7.8) | 99(7.5) | 21(9.6) | 0.287^c^ |
| Deep inferior epigastric perforator (DIEP) flap, no. (%) | 288(18.7) | 248(18.8) | 40(18.3) | 0.878^c^ | 277(19.1) | 239(19.2) | 38(18.4) | 0.784^c^ | 289(18.8) | 249(18.9) | 40(18.3) | 0.830^c^ |
| Latissimus dorsi (LD) flap, no. (%) | 49(3.2) | 46(3.5) | 3(1.4) | 0.101^c^ | 41(2.8) | 38(3.0) | 3(1.4) | 0.198^c^ | 49(3.2) | 46(3.5) | 3(1.4) | 0.098^c^ |
| Gluteal artery perforator (GAP) flap, no. (%) | 8(0.5) | 7(0.5) | 1(0.5) | 1^c^ | 8(0.6) | 7(0.6) | 1(0.5) | 1^c^ | 8(0.5) | 7(0.5) | 1(0.5) | 1^c^ |
| Superficial inferior epigastric artery (SIEA) flap, no. (%) | 48(3.1) | 48(3.6) | 0(0.00) | **0.004^c^** | 42(2.9) | 42(3.4) | 0(0.0) | **0.007^c^** | 48(3.1) | 48(3.6) | 0(0.0) | **0.004^c^** |
| Crossover flap, no. (%) | 59(3.8) | 51(3.9) | 8(3.7) | 0.890^c^ | 57(3.9) | 50(4.0) | 7(3.4) | 0.666^c^ | 59(3.8) | 51(3.9) | 8(3.7) | 0.879^c^ |
| Mixed flaps, no. (%) | 46(3.0) | 39(3.0) | 7(3.2) | 0.837^c^ | 41(2.8) | 36(2.9) | 5(2.4) | 0.704^c^ | 46(3.0) | 39(3.0) | 7(3.2) | 0.847^c^ |
| Mixed implant and autologous, no. (%) | 29(1.9) | 29(2.2) | 0(0.00) | 0.052^c^ | 28(1.9) | 28(2.2) | 0(0.0) | 0.057^c^ | 28(1.8) | 28(2.1) | 0(0.0) | 0.057^c^ |
| Chemotherapy^e^ |  |  |  | 0.410^c^ |  |  |  | 0.498^c^ |  |  |  | 0.398^c^ |
| Received, no. (%) | 438(28.5) | 381(28.9) | 57(26.1) |  | 415(28.5) | 360(28.9) | 55(26.6) |  | 437(28.4) | 380(28.8) | 57(26.0) |  |
| Not received, no. (%) | 1100(71.5) | 939(71.1) | 161(73.9) |  | 1039(71.5) | 887(71.1) | 152(73.4) |  | 1101(71.6) | 939(71.2) | 162(74.0) |  |
| Reconstruction laterality^e^ |  |  |  | 0.362^c^ |  |  |  | 0.298^c^ |  |  |  | 0.283^c^ |
| Unilateral, no. (%) | 697(45.3) | 592(44.8) | 105(48.2) |  | 654(45.0) | 554(44.4) | 100(48.3) |  | 693(45.1) | 587(44.5) | 106(48.4) |  |
| Bilateral, no. (%) | 841(54.7) | 728(55.2) | 113(51.8) |  | 800(55.0) | 693(55.6) | 107(51.7) |  | 845(54.9) | 732(55.5) | 113(51.6) |  |
| Mastectomy indication^e^ |  |  |  | 0.680^c^ |  |  |  | 0.397^c^ |  |  |  | 0.639^c^ |
| Therapeutic, no. (%) | 1385(90.1) | 1187(89.9) | 198(90.8) |  | 1311(90.2) | 1121(89.9) | 190(91.8) |  | 1384(90.0) | 1185(89.8) | 199(90.9) |  |
| Prophylactic, no. (%) | 153(9.9) | 133(10.1) | 20(9.2) |  | 143(9.8) | 126(10.1) | 17(8.2) |  | 154(10.0) | 134(10.2) | 20(9.1) |  |
| Axillary intervention^e^ |  |  |  |  |  |  |  |  |  |  |  |  |
| Axillary lymph node dissection (ALND), no. (%) | 397(25.8) | 354(26.8) | 43(19.7) | **0.027^c^** | 380(26.1) | 339(27.2) | 41(19.8) | **0.025^c^** | 395(25.7) | 352(26.7) | 43(19.6) | **0.027^c^** |
| Sentinel lymph node biopsy (SLNB), no. (%) | 691(44.9) | 579(43.9) | 112(51.4) | **0.039^c^** | 653(44.9) | 545(43.7) | 108(52.2) | **0.023^c^** | 692(45.0) | 579(43.9) | 113(51.6) | **0.034^c^** |
| None, no. (%) | 450(29.3) | 387(29.3) | 63(28.9) | 0.900^c^ | 421(29.0) | 363(29.1) | 58(28.0) | 0.749^c^ | 451(29.3) | 388(29.4) | 63(28.8) | 0.845^c^ |
| **Socioeconomic and ethnic data** |  |  |  |  |  |  |  |  |  |  |  |  |
| Marital status |  |  |  |  |  |  |  |  |  |  |  |  |
| Single, no. (%) | 107(7.0) | 98(7.5) | 9(4.1) | 0.073^c^ | 94(6.5) | 87(7.0) | 7(3.4) | 0.050^c^ | 107(7.0) | 98(7.5) | 9(4.1) | 0.071^c^ |
| Living with significant other, no. (%) | 67(4.4) | 59(4.5) | 8(3.7) | 0.580^c^ | 64(4.4) | 57(4.6) | 7(3.4) | 0.431^c^ | 67(4.4) | 59(4.5) | 8(3.7) | 0.572^c^ |
| Married, no. (%) | 1163(76.0) | 994(75.8) | 169(77.5) | 0.573^c^ | 1129(78.0) | 964(77.7) | 165(79.7) | 0.527^c^ | 1165(76.1) | 995(75.8) | 170(77.6) | 0.566^c^ |
| Separated, no. (%) | 26(1.7) | 22(1.7) | 4(1.8) | 1^c^ | 24(1.7) | 21(1.7) | 3(1.4) | 1^c^ | 26(1.7) | 22(1.7) | 4(1.8) | 1^c^ |
| Divorced, no. (%) | 125(8.2) | 104(7.9) | 21(9.6) | 0.394^c^ | 111(7.7) | 92(7.4) | 19(9.2) | 0.379^c^ | 124(8.1) | 103(7.9) | 21(9.6) | 0.383^c^ |
| Widowed, no. (%) | 42(2.7) | 35(2.7) | 7(3.2) | 0.649^c^ | 25(1.7) | 19(1.5) | 6(2.9) | 0.268^c^ | 42(2.7) | 35(2.7) | 7(3.2) | 0.657^c^ |
| Unknown, no. (%) | 8(0.5) | 8(0.6) | 0(0.0) | 0.519^c^ | 7(0.5) | 7(0.6) | 0(0.0) | 0.590^c^ | 7(0.5) | 7(0.5) | 0(0.0) | 0.590^c^ |
| Education level |  |  |  |  |  |  |  |  |  |  |  |  |
| Some high school, no. (%) | 31(2.0) | 29(2.2) | 2(0.9) | 0.321^c^ | 27(1.9) | 25(2.0) | 2(1.0) | 0.453^c^ | 31(2.0) | 29(2.2) | 2(0.9) | 0.318^c^ |
| High school degree, no. (%) | 122(8.0) | 113(8.6) | 9(4.1) | **0.024^c^** | 105(7.2) | 97(7.8) | 8(3.9) | **0.043^c^** | 121(7.9) | 112(8.5) | 9(4.1) | **0.025^c^** |
| Some college/trade school, no. (%) | 253(16.5) | 224(17.0) | 29(13.3) | 0.169^c^ | 236(16.3) | 210(16.9) | 26(12.6) | 0.119^c^ | 252(16.4) | 223(16.9) | 29(13.2) | 0.171^c^ |
| College/trade school degree, no. (%) | 597(38.9) | 514(39.1) | 83(38.1) | 0.776^c^ | 572(39.4) | 493(39.6) | 79(38.2) | 0.689^c^ | 596(38.8) | 512(38.9) | 84(38.4) | 0.877^c^ |
| Some masters/doctoral, no. (%) | 60(3.9) | 53(4.0) | 7(3.2) | 0.563^c^ | 57(3.9) | 51(4.1) | 6(2.9) | 0.410^c^ | 60(3.9) | 53(4.0) | 7(3.2) | 0.557^c^ |
| Masters/doctoral degree, no. (%) | 470(30.7) | 382(29.0) | 88(40.4) | **0.001^c^** | 454(31.3) | 368(29.6) | 86(41.5) | **0.001^c^** | 475(30.9) | 387(29.4) | 88(40.2) | **0.001^c^** |
| Unknown, no. (%) | 5(0.3) | 5(0.4) | 0(0.0) | 0.789^c^ | 3(0.2) | 3(0.2) | 0(0.0) | 1^c^ | 3(0.2) | 3(0.2) | 0(0.0) | 1^c^ |
| Working status |  |  |  |  |  |  |  |  |  |  |  |  |
| Unable to work, no. (%) | 37(2.4) | 29(2.2) | 8(3.7) | 0.196^c^ | 33(2.3) | 26(2.1) | 7(3.4) | 0.372^c^ | 37(2.4) | 29(2.2) | 8(3.7) | 0.200^c^ |
| Unemployed, no. (%) | 32(2.1) | 29(2.2) | 3(1.4) | 0.585^c^ | 29(2.0) | 26(2.1) | 3(1.5) | 0.727^c^ | 31(2.0) | 28(2.1) | 3(1.4) | 0.626^c^ |
| Student, no. (%) | 10(0.7) | 9(0.7) | 1(0.5) | 1^c^ | 9(0.6) | 9(0.7) | 0(0.0) | 0.452^c^ | 10(0.7) | 9(0.7) | 1(0.5) | 1^c^ |
| Volunteer, no. (%) | 8(0.5) | 7(0.5) | 1(0.5) | 1^c^ | 7(0.5) | 6(0.5) | 1(0.5) | 1^c^ | 8(0.5) | 7(0.5) | 1(0.5) | 1^c^ |
| Retired, no. (%) | 139(9.1) | 128(9.8) | 11(5.1) | **0.024^c^** | 117(8.1) | 108(8.8) | 9(4.4) | **0.033^c^** | 140(9.2) | 129(9.9) | 11(5.0) | **0.022^c^** |
| Homemaker, no. (%) | 177(11.6) | 151(11.6) | 26(12.0) | 0.867^c^ | 172(12.0) | 146(11.8) | 26(12.6) | 0.749^c^ | 176(11.6) | 150(11.5) | 26(11.9) | 0.856^c^ |
| Part time employed, no. (%) | 216(14.2) | 175(13.4) | 41(18.9) | **0.033^c^** | 204(14.2) | 164(13.3) | 40(19.4) | **0.020^c^** | 215(14.1) | 174(13.3) | 41(18.8) | **0.032^c^** |
| Full time employed, no. (%) | 849(55.9) | 734(56.3) | 115(53.0) | 0.359^c^ | 818(56.8) | 709(57.5) | 109(52.9) | 0.218^c^ | 853(56.0) | 737(56.5) | 116(53.2) | 0.362^c^ |
| Other, no. (%) | 52(3.4) | 41(3.1) | 11(5.1) | 0.149^c^ | 50(3.5) | 39(3.2) | 11(5.3) | 0.114^c^ | 52(3.4) | 41(3.1) | 11(5.0) | 0.153^c^ |
| Unknown, no. (%) | 18(1.2) | 17(1.3) | 1(0.5) | 0.475^c^ | 15(1.0) | 14(1.1) | 1(0.5) | 0.637^c^ | 16(1.0) | 15(1.1) | 1(0.5) | 0.576^c^ |
| Household income per year |  |  |  |  |  |  |  |  |  |  |  |  |
| <25,000$, no. (%) | 80(5.4) | 73(5.8) | 7(3.3) | 0.137^c^ | 68(4.8) | 62(5.1) | 6(2.9) | 0.174^c^ | 80(5.4) | 73(5.7) | 7(3.3) | 0.134^c^ |
| 25,000$ to 49,999$, no. (%) | 161(10.9) | 147(11.6) | 14(6.5) | **0.028^c^** | 145(10.3) | 133(11.0) | 12(5.9) | **0.025^c^** | 160(10.8) | 146(11.5) | 14(6.5) | **0.029^c^** |
| 50,000$ to 74,999$, no. (%) | 265(17.9) | 229(18.0) | 36(16.8) | 0.666^c^ | 244(17.3) | 212(17.6) | 32(15.7) | 0.506^c^ | 263(17.7) | 227(17.9) | 36(16.7) | 0.688^c^ |
| 75,000$ to 99,999$, no. (%) | 235(15.8) | 209(16.5) | 26(12.1) | 0.109^c^ | 223(15.8) | 197(16.3) | 26(12.7) | 0.192^c^ | 233(15.7) | 207(16.3) | 26(12.1) | 0.117^c^ |
| >100,000$, no. (%) | 742(50.0) | 611(48.1) | 131(61.2) | **0.0004^c^** | 729(51.7) | 601(49.9) | 128(62.7) | **0.001^c^** | 749(50.4) | 617(48.6) | 132(61.4) | **0.001^c^** |
| Unknown, no. (%) | 55(3.6) | 51(3.9) | 4(1.8) | 0.135^c^ | 45(3.1) | 42(3.4) | 3(1.4) | 0.140^c^ | 53(3.4) | 49(3.7) | 4(1.8) | 0.156^c^ |
| **Race background** |  |  |  |  |  |  |  |  |  |  |  |  |
| Caucasian, no. (%) | 1385(90.9) | 1187(90.7) | 198(92.1) | 0.505^c^ | 1317(91.3) | 1129(91.1) | 188(92.2) | 0.628^c^ | 1386(90.8) | 1188(90.7) | 198(91.7) | 0.644^c^ |
| Asian, no. (%) | 60(3.9) | 53(4.0) | 7(3.3) | 0.579^c^ | 56(3.9) | 49(4.0) | 7(3.4) | 0.720^c^ | 61(4.0) | 53(4.0) | 8(3.7) | 0.812^c^ |
| African American, no. (%) | 68(4.5) | 60(4.6) | 8(3.7) | 0.570^c^ | 59(4.1) | 52(4.2) | 7(3.4) | 0.609^c^ | 68(4.5) | 60(4.6) | 8(3.7) | 0.563^c^ |
| American Indian/Alaska Native, no. (%) | 8(0.5) | 7(0.5) | 1(0.5) | 1^c^ | 8(0.6) | 7(0.6) | 1(0.5) | 1^c^ | 8(0.5) | 7(0.5) | 1(0.5) | 1^c^ |
| Native Hawaiian/Other Pacific Islander, no. (%) | 3(0.20) | 2(0.2) | 1(0.5) | 0.899^c^ | 3(0.2) | 2(0.2) | 1(0.5) | 0.900^c^ | 3(0.2) | 2(0.2) | 1(0.5) | 0.901^c^ |
| Unknown, no. (%) | 14(0.9) | 11(0.8) | 3(1.4) | 0.691^c^ | 11(0.8) | 8(0.6) | 3(1.4) | 0.419^c^ | 12(0.8) | 9(0.7) | 3(1.4) | 0.512^c^ |
| **Outcome— patient-reported well-being at 2-year follow-up compared to baseline^e^** |  |  |  |  |  |  |  |  |  |  |  |  |
| Improved^d^, no. (%) | 563(36.6) | 491(37.2) | 72(33.0) | 0.236^c^ | 592(40.7) | 517(41.5) | 75(36.2) | 0.156^c^ | 769(50.0) | 662(50.2) | 107(48.9) | 0.715^c^ |
| Worsened^d^, no. (%) | 737(47.9) | 630(47.7) | 107(49.1) | 0.711^c^ | 647(44.5) | 555(44.5) | 92(44.4) | 0.987^c^ | 453(29.5) | 392(29.7) | 61(27.9) | 0.575^c^ |
| Stable, no. (%) | 238(15.5) | 199(15.1) | 39(17.9) | 0.287^c^ | 215(14.8) | 175(14.0) | 40(19.3) | **0.047^c^** | 316(20.5) | 265(20.1) | 51(23.3) | 0.278^c^ |

Note:*P* values < 0.05 highlighted in bold.

^a^*P* values refer to differences in the development and validation set.

^b^*P* values refer to t-tests to evaluate mean differences of continuous data.

^c^*P* values refer to Chi-square tests for binary feature evaluation (feature true vs. feature not true).

^d^ Increase or decrease at least by minimal clinically important difference compared to baseline (3 for physical well-being, 4 for both sexual and psychosocial well-beings in this study) ^1^.

^e^ Variable included in the predictive models.

**Reference**

1. Voineskos SH, Klassen AF, Cano SJ, Pusic AL, Gibbons CJ. Giving Meaning to Differences in BREAST-Q Scores: Minimal Important Difference for Breast Reconstruction Patients. *Plast Reconstr Surg*. 2020;145(1):11e-20e. doi:10.1097/PRS.0000000000006317
