# Supplementary material for: Enhanced Surgical Decision-Making Tools in Breast Cancer: Predicting 2-Year Postoperative Physical, Sexual, and Psychosocial Well-Being following Mastectomy and Breast Reconstruction (INSPiRED 004)
Source: Ann Surg Oncol. 2023 Jul 30;30(12):7046–59. doi: 10.1245/s10434-023-13971-w (PMC10562277; doi:10.1245/s10434-023-13971-w)

**Supplement 3**

**Figure 1. Calibration Plots of Machine Learning Models to Predict Physical Well-being with Reconstructed Breasts**

(A) Worsened physical well-being- Logistic Regression with Elastic Net Penalty (B) Worsened physical well-being-XGBoost Tree

(C) Worsened physical well-being-Neural Network (D) Improved physical well-being-Logistic Regression with Elastic Net Penalty

(E) Improved physical well-being-XGBoost Tree (F) Improved physical well-being-Neural Network


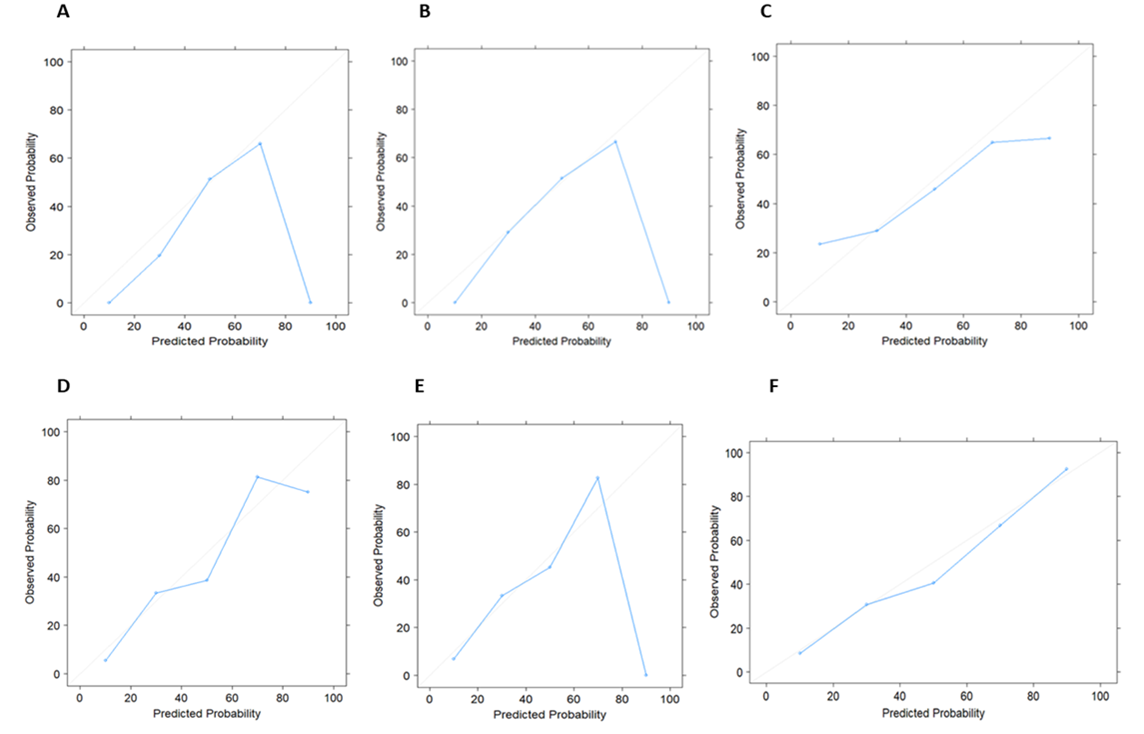


**Figure 2. Calibration Plots of Machine Learning Models to Predict Sexual Well-being with Reconstructed Breasts**

(A)Worsened sexual well-being- Logistic Regression with Elastic Net Penalty (B)Worsened sexual well-being-XGBoost Tree

(C)Worsened sexual well-being-Neural Network (D)Improved sexual well-being-Logistic Regression with Elastic Net Penalty

(E)Improved sexual well-being-XGBoost Tree (F)Improved sexual well-being-Neural Network


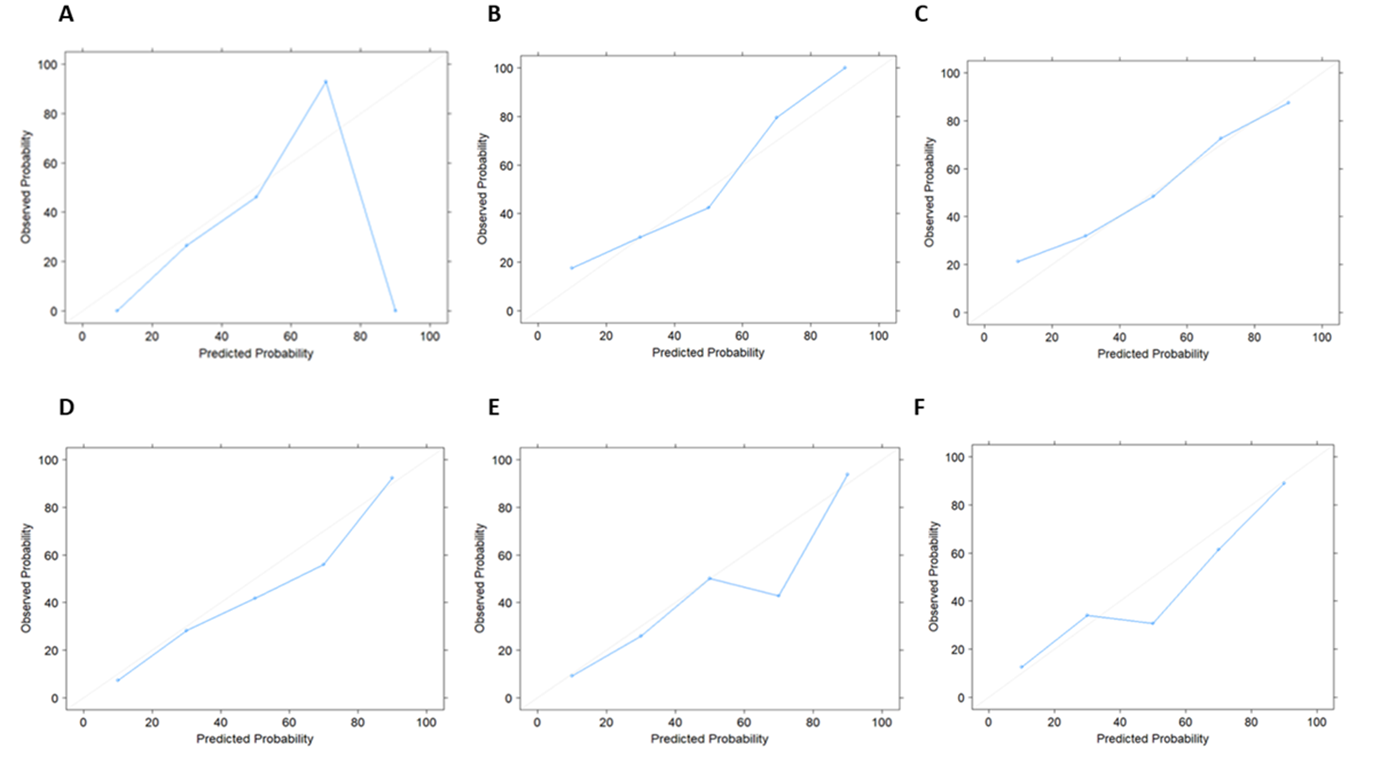


**Figure 3. Calibration Plots of Machine Learning Models to Predict Psychosocial Well-being with Reconstructed Breasts**

(A) Worsened psychosocial well-being- Logistic Regression with Elastic Net Penalty (B) Worsened psychosocial well-being-XGBoost Tree

(C) Worsened psychosocial well-being-Neural Network (D) Improved psychosocial well-being-Logistic Regression with Elastic Net Penalty

(E) Improved psychosocial well-being-XGBoost Tree (F) Improved psychosocial well-being-Neural Network


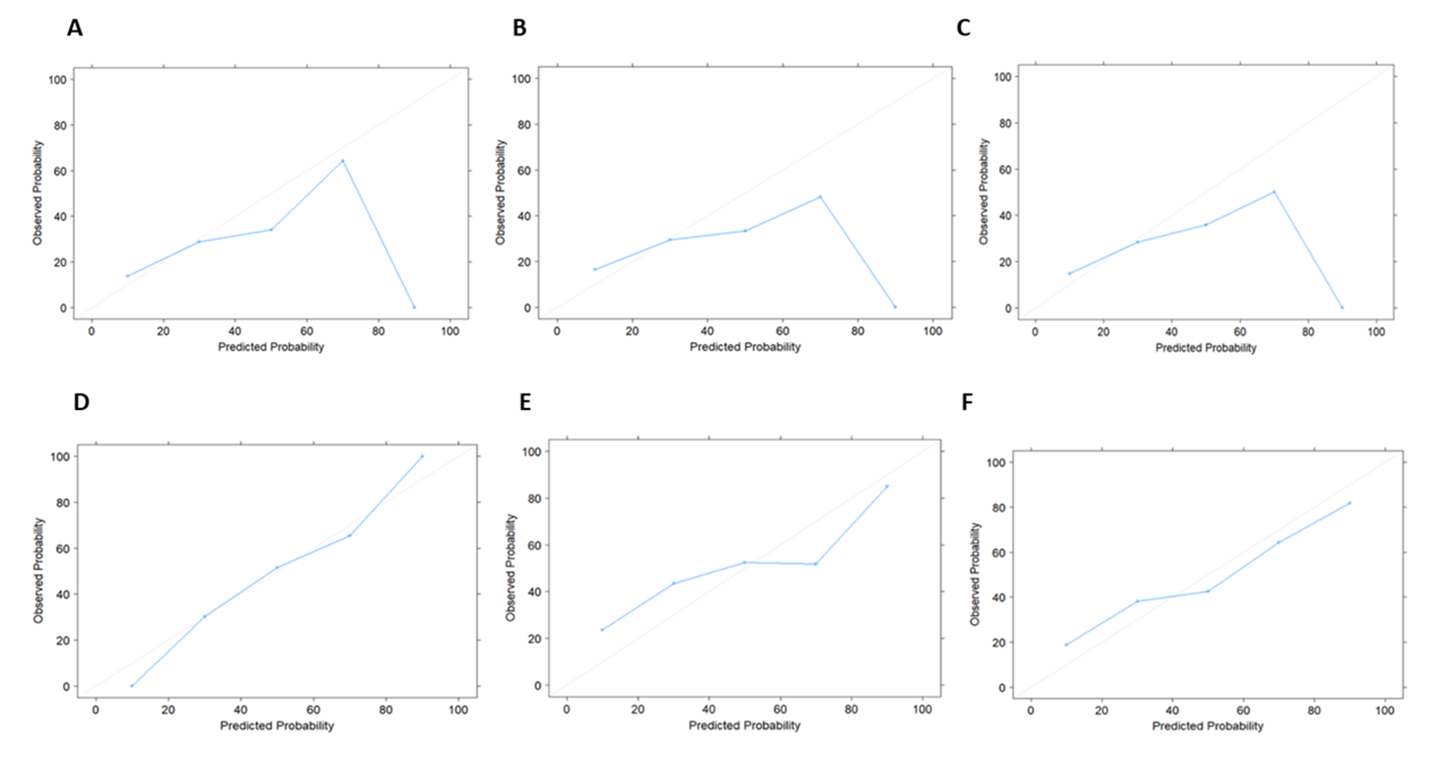


**Figure 4. Shapley Additive Explanations (SHAP) Value Summary Plot of the Extreme Gradient Boosting (XGBoost) Tree Model**

(A)Worsened physical well-being(B)Improved physical well-being

(C)Worsened sexual well-being(D)Improved sexual well-being

(E)Worsened psychosocial well-being(F)Improved psychosocial well-being


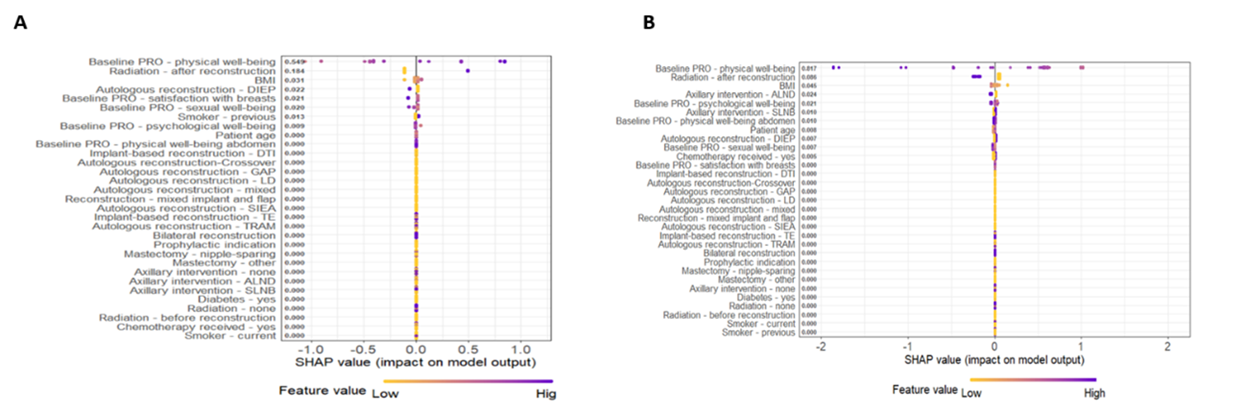


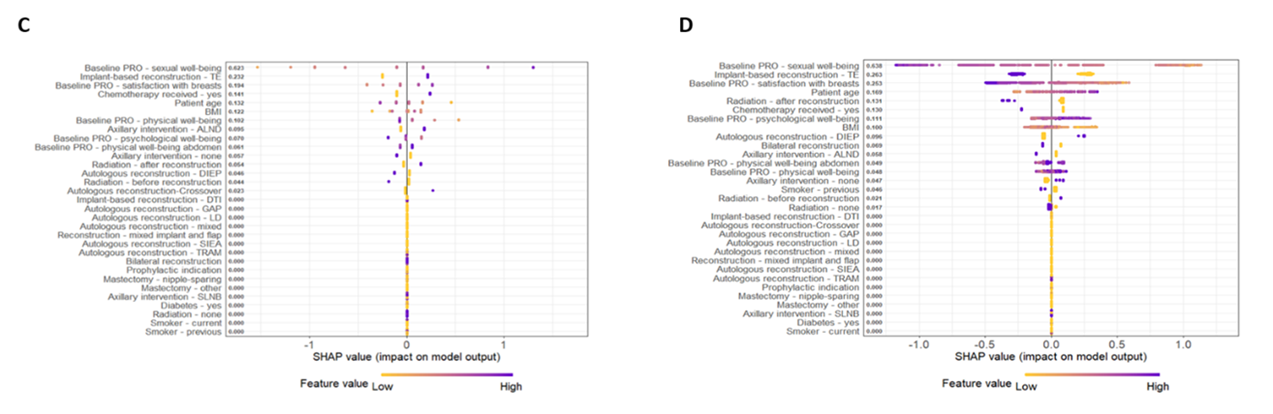


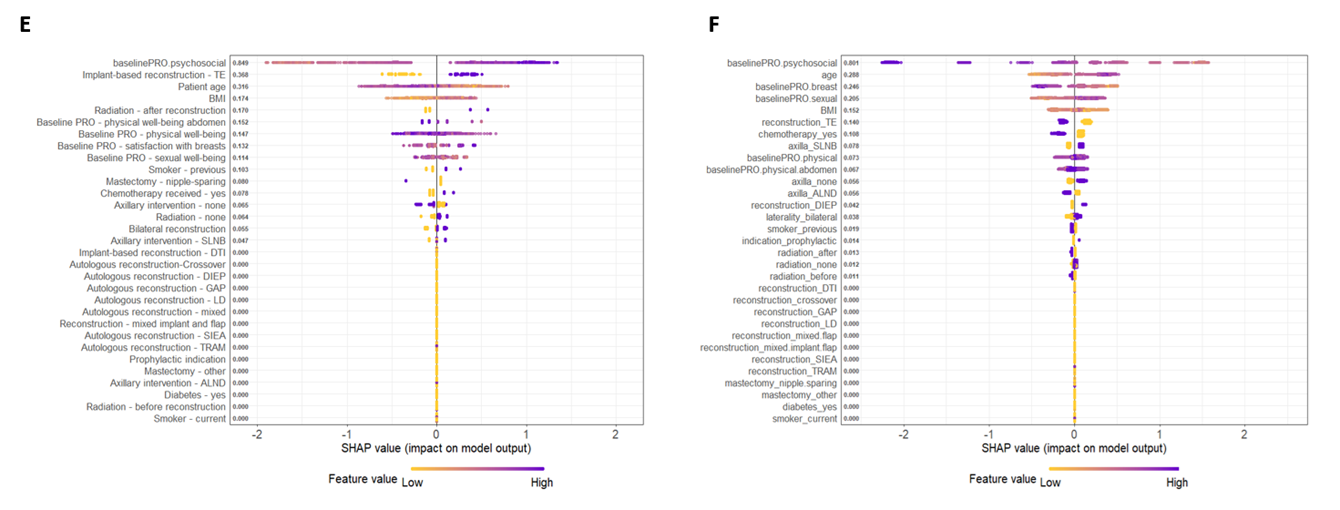


**Figure 5. Local Interpretable Model-agnostic Explanations of the Neural Network**

(A)Worsened physical well-being (B)Improved physical well-being

(C)Worsened sexual well-being (D)Improved sexual well-being

(E)Worsened psychosocial well-being (F)Improved psychosocial well-being


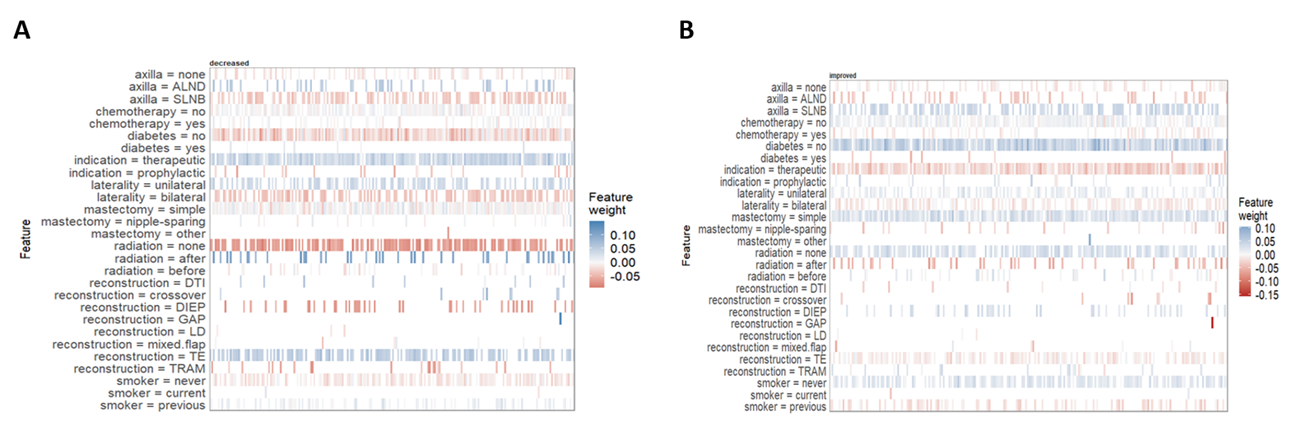


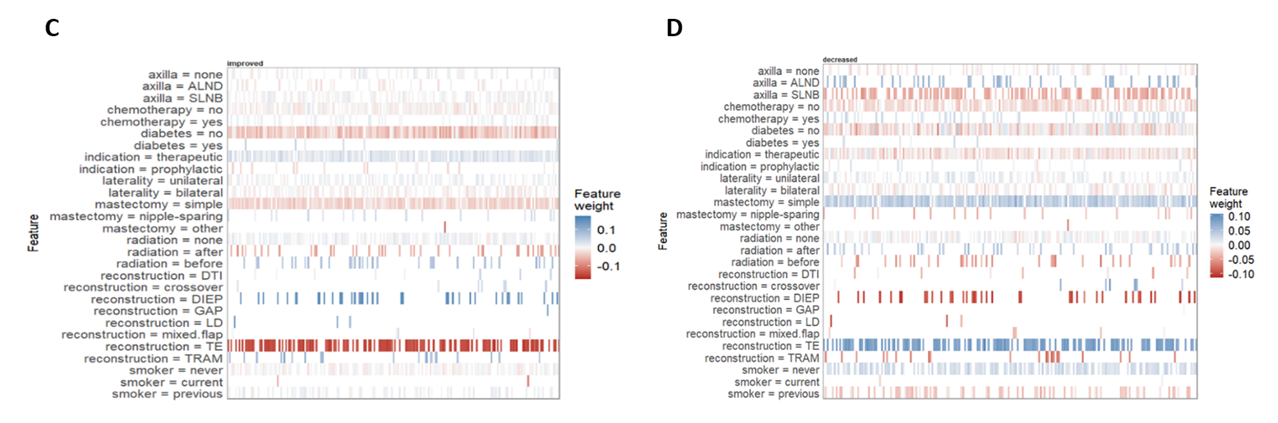


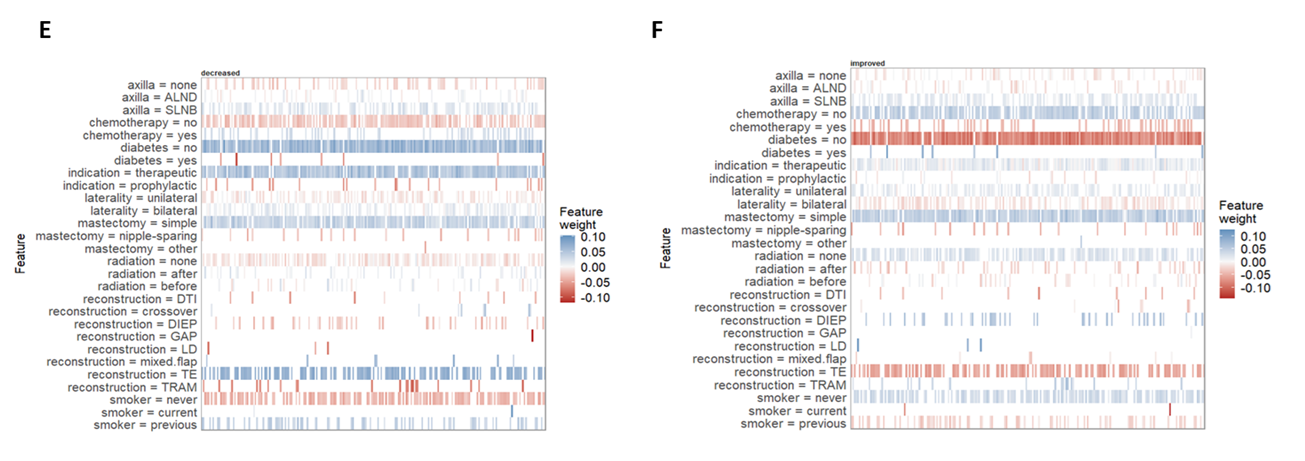

Supplement: Supplementary file 3 — Supplementary file3 (DOCX 2235 KB) [file 10434_2023_13971_MOESM3_ESM.docx]
