# Supplementary material for: Enhanced Surgical Decision-Making Tools in Breast Cancer: Predicting 2-Year Postoperative Physical, Sexual, and Psychosocial Well-Being following Mastectomy and Breast Reconstruction (INSPiRED 004)
Source: Ann Surg Oncol. 2023 Jul 30;30(12):7046–59. doi: 10.1245/s10434-023-13971-w (PMC10562277; doi:10.1245/s10434-023-13971-w)
Supplement: Supplementary file 4 — Supplementary file4 (DOCX 48 KB) [file 10434_2023_13971_MOESM4_ESM.docx]

**Supplement 4**

**Table 1**. **Baseline Characteristics Comparison between Patients Followed for 2 Years and Patients Lost to Follow-Up**

|  | **Physical well-being (chest and upper body)** | | | **Sexual well-being** | | | **Psychosocial well-being** | | |
| --- | --- | --- | --- | --- | --- | --- | --- | --- | --- |
|  | Patients followed for 2 years | patients lost to follow up | *P* value^a^ | Patients followed for 2 years | Patients lost to follow-up | *P* value^a^ | Patients followed for 2 years | Patients lost to follow-up | *P* value^a^ |
|  | (n=1538) | (n=1511) |  | (n=1454) | (n=1523) |  | (n=1538) | (n=1507) |  |
| **Patient variables** |  |  |  |  |  |  |  |  |  |
| Age, mean (SD), years | 50.25(10) | 48.79 (10.23) | **<0.01^b^** | 49.86 (9.87) | 48.85 (10.18) | **0.01^b^** | 50.19 (9.97) | 48.82 (10.26) | **0.0002^b^** |
| BMI, mean (SD), kg/m^2^ | 26.5 (5.4) | 27.13 (6.06) | **0.002^b^** | 26.4 (5.35) | 27.19 (6.03) | **0.0001^b^** | 26.48 (5.39) | 27.15 (6.07) | **0.001^b^** |
| Diabetes, no (%) |  |  | 0.61^c^ |  |  | 0.93^c^ |  |  | 0.62^c^ |
| No, no. (%) | 1467(95.4) | 1447(95.8) |  | 1391(95.7) | 1456(95.6) |  | 1467(95.4) | 1443(95.8) |  |
| Yes, no. (%) | 71(4.6) | 64(4.2) |  | 63(4.3) | 67(4.4) |  | 71(4.6) | 64(4.2) |  |
| Smoker |  |  |  |  |  |  |  |  |  |
| Never, no. (%) | 1015(66.6) | 953(63.7) | 0.10^c^ | 971(67.3) | 955(63.4) | **0.02^c^** | 1020(66.9) | 947(63.6) | 0.06^c^ |
| Previous, no. (%) | 483(31.7) | 495(33.1) | 0.41^c^ | 446(30.9) | 503(33.4) | 0.15^c^ | 479(31.4) | 494(33.2) | 0.31^c^ |
| Current, no. (%) | 27(1.8) | 48(3.2) | **0.01^c^** | 25(1.7) | 49(3.3) | **0.01^c^** | 26(1.7) | 49(3.3) | **0.01^c^** |
| Unknown, no. (%) | 13(0.8) | 15(1.0) | 0.67^c^ | 12(0.8) | 16(1.1) | 0.52^c^ | 13(0.8) | 17(1.1) | 0.43^c^ |
| **Pre-operative patient-reported outcome data** |  |  |  |  |  |  |  |  |  |
| BREAST-Q satisfaction with breast, mean (SD), 0-100 | 60.06 (22.17) | 58.93 (22.19) | 0.16^b^ | 60.25 (22.2) | 58.61 (22.11) | **0.04^b^** | 60.07 (22.13) | 58.87 (22.22) | 0.14^b^ |
| BREAST-Q physical well-being chest and upper body, mean (SD), 0-100 | 78.85 (14.54) | 78.76 (15.16) | 0.86^b^ | 78.99 (14.36) | 78.67 (15.12) | 0.56^b^ | 78.85(14.54) | 78.77(15.16) | 0.88^b^ |
| BREAST-Q psychosocial well-being, mean (SD), 0-100 | 69.64 (18.13) | 68.68 (18.62) | 0.15^b^ | 69.53 (18.09) | 68.58 (18.58) | 0.15^b^ | 69.62(18.09) | 68.67 (18.67) | 0.15^b^ |
| BREAST-Q physical well-being abdomen, mean (SD), 0-100 | 89.51 (13.49) | 89.58 (14.21) | 0.89^b^ | 89.6 (13.29) | 89.44 (14.26) | 0.75^b^ | 89.51 (13.49) | 89.56 (14.26) | 0.93^b^ |
| BREAST-Q sexual well-being, mean (SD), 0-100 | 54.85 (20.74) | 54.6 (20.61) | 0.74^b^ | 55.37 (20.44) | 54.07 (20.9) | 0.08^b^ | 54.94 (20.72) | 54.49 (20.63) | 0.56^b^ |
| **Clinical variables** |  |  |  |  |  |  |  |  |  |
| Radiation |  |  |  |  |  |  |  |  |  |
| After reconstruction, no. (%) | 287(18.7) | 329(21.8) | **0.03^c^** | 274(18.8) | 329(21.6) | 0.06^c^ | 287(18.7) | 330(21.9) | **0.03^c^** |
| Before reconstruction, no. (%) | 224(14.6) | 218(14.4) | 0.91^c^ | 201(13.8) | 228(15.0) | 0.37^c^ | 220(14.3) | 219(14.5) | 0.86^c^ |
| None, no. (%) | 1027(66.8) | 964(63.8) | 0.08^c^ | 979(67.3) | 966(63.4) | **0.03^c^** | 1031(67.0) | 958(63.6) | **0.04^c^** |
| Mastectomy |  |  |  |  |  |  |  |  |  |
| Nipple-sparing, no. (%) | 166(10.8) | 212(14.0) | **0.01^c^** | 162(11.1) | 211(13.9) | **0.03^c^** | 168(10.9) | 211(14.0) | **0.01^c^** |
| Simple, no. (%) | 1366(88.8) | 1288(85.2) | **0.003^c^** | 1286(88.4) | 1301(85.4) | **0.02^c^** | 1364(88.7) | 1285(85.3) | **0.01^c^** |
| Other, no. (%) | 6(0.4) | 11(0.7) | 0.21^c^ | 6(0.4) | 11(0.7) | 0.26^c^ | 6(0.4) | 11(0.7) | 0.21^c^ |
| Reconstruction technique |  |  |  |  |  |  |  |  |  |
| Tissue expander (TE), no. (%) | 820(53.3) | 1000(66.2) | **<0.001^c^** | 778(53.5) | 1002(65.8) | **<0.001^c^** | 820(53.3) | 998(66.2) | **<0.001^c^** |
| Direct-to-implant (DTI), no. (%) | 71(4.6) | 59(3.9) | 0.33^c^ | 67(4.6) | 58(3.8) | 0.28^c^ | 71(4.6) | 59(3.9) | 0.34^c^ |
| Transverse rectus abdominis (TRAM) flap, no. (%) | 120(7.8) | 92(6.1) | 0.06^c^ | 115(7.9) | 93(6.1) | 0.05^c^ | 120(7.8) | 92(6.1) | 0.07^c^ |
| Deep inferior epigastric perforator (DIEP) flap, no. (%) | 288(18.7) | 189(12.5) | **<0.001^c^** | 277(19.1) | 188(12.3) | **<0.001^c^** | 289(18.8) | 186(12.3) | **<0.001^c^** |
| Latissimus dorsi (LD) flap, no. (%) | 49(3.2) | 31(2.1) | 0.05^c^ | 41(2.8) | 36(2.4) | 0.43^c^ | 49(3.2) | 31(2.1) | 0.05^c^ |
| Gluteal artery perforator (GAP) flap, no. (%) | 8(0.5) | 6(0.4) | 0.62^c^ | 8(0.6) | 6(0.4) | 0.53^c^ | 8(0.5) | 6(0.4) | 0.62^c^ |
| Superficial inferior epigastric artery (SIEA) flap, no. (%) | 48(3.1) | 25(1.7) | **0.01^c^** | 42(2.9) | 28(1.8) | 0.06^c^ | 48(3.1) | 25(1.7) | **0.01^c^** |
| Crossover flap, no. (%) | 59(3.8) | 47(3.1) | 0.27^c^ | 57(3.9) | 48(3.2) | 0.26^c^ | 59(3.8) | 47(3.1) | 0.28^c^ |
| Mixed flaps, no. (%) | 29(1.9) | 27(1.8) | 0.84^c^ | 28(1.9) | 27(1.8) | 0.76^c^ | 28(1.8) | 28(1.9) | 0.94^c^ |
| Mixed implant and autologous, no. (%) | 46(3.0) | 35(2.3) | 0.25^c^ | 41(2.8) | 37(2.4) | 0.51^c^ | 46(3.0) | 35(2.3) | 0.25^c^ |
| Chemotherapy |  |  | 0.12^c^ |  |  | 0.21^c^ |  |  | 0.10^c^ |
| Received, no. (%) | 438(28.5) | 469(31.0) |  | 415(28.5) | 467(30.7) |  | 437(28.4) | 469(31.1) |  |
| Not received, no. (%) | 1100(71.5) | 1042(69.0) |  | 1039(71.5) | 1056(69.3) |  | 1101(71.6) | 1038(68.9) |  |
| Reconstruction laterality |  |  | 0.07^c^ |  |  | 0.15^c^ |  |  | 0.10^c^ |
| Unilateral, no. (%) | 697(45.3) | 636(42.1) |  | 654(45.0) | 645(42.4) |  | 693(45.1) | 635(42.1) |  |
| Bilateral, no. (%) | 841(54.7) | 875(57.9) |  | 800(55.0) | 878(57.6) |  | 845(54.9) | 872(57.9) |  |
| Mastectomy indication |  |  | 0.20^c^ |  |  | 0.13^c^ |  |  | 0.23^c^ |
| Therapeutic, no. (%) | 1385(90.1) | 1339(88.6) |  | 1311(90.2) | 1347(88.4) |  | 1384(90.0) | 1336(88.7) |  |
| Prophylactic, no. (%) | 153 (9.9) | 172(11.4) |  | 143(9.8) | 176(11.6) |  | 154(10.0) | 171(11.3) |  |
| Axillary intervention |  |  |  |  |  | 0.44^c^ |  |  |  |
| Axillary lymph node dissection (ALND), no. (%) | 397(25.8) | 414(27.4) | 0.32^c^ | 380(26.1) | 417(27.4) |  | 395(25.7) | 415(27.5) | 0.25^c^ |
| Sentinel lymph node biopsy (SLNB), no. (%) | 691(44.9) | 675(44.7) | 0.89^c^ | 653(44.9) | 674(44.3) | 0.72^c^ | 692(45.0) | 671(44.5) | 0.80^c^ |
| None, no. (%) | 450(29.3) | 422(27.9) | 0.42^c^ | 421(29.0) | 432(28.4) | 0.72^c^ | 451(29.3) | 421(27.9) | 0.40^c^ |
| **Socioeconomic and ethnic data** |  |  |  |  |  |  |  |  |  |
| Marital status |  |  |  |  |  |  |  |  |  |
| Single, no. (%) | 107(7.0) | 152(10.1) | **0.001^c^** | 94(6.5) | 151(10.0) | **0.001^c^** | 107(7.0) | 153(10.3) | **0.001^c^** |
| Living with significant other, no. (%) | 67(4.4) | 80(5.3) | 0.22^c^ | 64(4.4) | 81(5.4) | 0.23^c^ | 67(4.4) | 80(5.4) | 0.21^c^ |
| Married, no. (%) | 1163(76.0) | 1047(69.8) | **0.0001^c^** | 1129(78.0) | 1054(69.9) | **<0.001^c^** | 1165(76.1) | 1040(69.7) | **<0.001^c^** |
| Separated, no. (%) | 26(1.7) | 36(2.4) | 0.17^c^ | 24(1.7) | 35(2.3) | 0.20^c^ | 26(1.7) | 36(2.4) | 0.17^c^ |
| Divorced, no. (%) | 125(8.2) | 152(10.1) | 0.06^c^ | 111(7.7) | 149(9.9) | **0.03^c^** | 124(8.1) | 151(10.1) | 0.05^c^ |
| Widowed, no. (%) | 42(2.7) | 32(2.1) | 0.28^c^ | 25(1.7) | 38(2.5) | 0.14^c^ | 42(2.7) | 32(2.1) | 0.29^c^ |
| Unknown, no. (%) | 8(0.5) | 12(0.8) | 0.35^c^ | 7(0.5) | 15(1.0) | 0.11^c^ | 7(0.5) | 15(1.0) | 0.08^c^ |
| Education level |  |  |  |  |  |  |  |  |  |
| Some high school, no. (%) | 31(2.0) | 33(2.2) | 0.74^c^ | 27(1.9) | 34(2.2) | 0.46^c^ | 31(2.0) | 33(2.2) | 0.72^c^ |
| High school degree, no. (%) | 122(8.0) | 122(8.1) | 0.87^c^ | 105(7.2) | 128(8.5) | 0.21^c^ | 121(7.9) | 123(8.2) | 0.73^c^ |
| Some college/trade school, no. (%) | 253(16.5) | 257(17.1) | 0.65^c^ | 236(16.3) | 256(16.9) | 0.63^c^ | 252(16.4) | 256(17.1) | 0.61^c^ |
| College/trade school degree, no. (%) | 597(38.9) | 588(39.1) | 0.91^c^ | 572(39.4) | 592(39.2) | 0.88^c^ | 596(38.8) | 588(39.3) | 0.79^c^ |
| Some masters/doctoral, no. (%) | 60(3.9) | 84(5.6) | **0.03^c^** | 57(3.9) | 84(5.6) | **0.04^c^** | 60(3.9) | 84(5.6) | **0.03^c^** |
| Masters/doctoral degree, no. (%) | 470(30.7) | 418(27.8) | 0.09^c^ | 454(31.3) | 418(27.6) | **0.03^c^** | 475(30.9) | 412(27.5) | **0.04^c^** |
| Unknown, no. (%) | 5(0.3) | 9(0.6) | 0.27^c^ | 3(0.2) | 11(0.7) | 0.07^c^ | 3(0.2) | 11(0.7) | 0.06^c^ |
| Working status |  |  |  |  |  |  |  |  |  |
| Unable to work, no. (%) | 37(2.4) | 53(3.6) | 0.07^c^ | 33(2.3) | 52(3.5) | 0.06^c^ | 37(2.4) | 53(3.6) | 0.07^c^ |
| Unemployed, no. (%) | 32(2.1) | 34(2.3) | 0.74^c^ | 29(2.0) | 35(2.3) | 0.55^c^ | 31(2.0) | 35(2.4) | 0.55^c^ |
| Student, no. (%) | 10(0.7) | 16(1.1) | 0.22^c^ | 9(0.6) | 16(1.1) | 0.19^c^ | 10(0.7) | 16(1.1) | 0.21^c^ |
| Volunteer, no. (%) | 8(0.5) | 9(0.6) | 0.78^c^ | 7(0.5) | 10(0.7) | 0.52^c^ | 8(0.5) | 9(0.6) | 0.77^c^ |
| Retired, no. (%) | 139(9.1) | 107(7.2) | 0.05^c^ | 117(8.1) | 107(7.1) | 0.31^c^ | 140(9.2) | 106(7.1) | **0.04^c^** |
| Homemaker, no. (%) | 177(11.6) | 170(11.4) | 0.84^c^ | 172(12.0) | 170(11.3) | 0.60^c^ | 176(11.6) | 171(11.5) | 0.97^c^ |
| Part time employed, no. (%) | 216(14.2) | 166(11.1) | **0.01^c^** | 204(14.2) | 171(11.4) | **0.02^c^** | 215(14.1) | 165(11.1) | **0.01^c^** |
| Full time employed, no. (%) | 849(55.9) | 869(58.3) | 0.17^c^ | 818(56.8) | 874(58.3) | 0.44^c^ | 853(56.0) | 863(58.2) | 0.24^c^ |
| Other, no. (%) | 52(3.4) | 66(4.4) | 0.15^c^ | 50(3.5) | 65(4.3) | 0.23^c^ | 52(3.4) | 66(4.4) | 0.15^c^ |
| Unknown, no. (%) | 18(1.2) | 21(1.4) | 0.59^c^ | 15(1.0) | 23(1.5) | 0.24^c^ | 16(1.0) | 23(1.5) | 0.23^c^ |
| Household income per year |  |  |  |  |  |  |  |  |  |
| <25,000$, no. (%) | 80(5.4) | 109(7.4) | **0.03^c^** | 68(4.8) | 109(7.3) | **0.005^c^** | 80(5.4) | 109(7.4) | **0.02^c^** |
| 25,000$ to 49,999$, no. (%) | 161(10.9) | 179(12.1) | 0.27^c^ | 145(10.3) | 181(12.2) | 0.10^c^ | 160(10.8) | 180(12.3) | 0.21^c^ |
| 50,000$ to 74,999$, no. (%) | 265(17.9) | 252(17.1) | 0.58^c^ | 244(17.3) | 253(17.1) | 0.85^c^ | 263(17.7) | 253(17.2) | 0.73^c^ |
| 75,000$ to 99,999$, no. (%) | 235(15.8) | 224(15.2) | 0.63^c^ | 223(15.8) | 233(15.7) | 0.93^c^ | 233(15.7) | 224(15.3) | 0.75^c^ |
| >100,000$, no. (%) | 742(50.0) | 710(48.2) | 0.31^c^ | 729(51.7) | 707(47.7) | **0.03^c^** | 749(50.4) | 702(47.8) | 0.15^c^ |
| Unknown, no. (%) | 55(3.6) | 37(2.4) | 0.07^c^ | 45(3.1) | 40(2.6) | 0.44^c^ | 53(3.4) | 39(2.6) | 0.17^c^ |
| **Race background** |  |  |  |  |  |  |  |  |  |
| Caucasian, no. (%) | 1385(90.9) | 1262(84.6) | **<0.001^c^** | 1317(91.3) | 1273(84.9) | **<0.001^c^** | 1386(90.8) | 1260(84.9) | **<0.001^c^** |
| Asian, no. (%) | 60(3.9) | 83(5.6) | **0.04^c^** | 56(3.9) | 83(5.5) | **0.03^c^** | 61(4.0) | 81(5.5) | 0.06^c^ |
| African American, no. (%) | 68(4.5) | 124(8.3) | **<0.001^c^** | 59(4.1) | 121(8.1) | **<0.001^c^** | 68(4.5) | 121(8.2) | **<0.001^c^** |
| American Indian/Alaska Native, no. (%) | 8(0.5) | 20(1.3) | **0.02^c^** | 8(0.6) | 20(1.3) | **0.03^c^** | 8(0.5) | 20(1.3) | **0.02^c^** |
| Native Hawaiian/Other Pacific Islander, no. (%) | 3(0.2) | 2(0.1) | 1^c^ | 3(0.2) | 2(0.1) | 0.97^c^ | 3(0.2) | 2(0.1) | 1^c^ |
| Unknown, no. (%) | 14(0.9) | 20(1.3) | 0.36^c^ | 11(0.8) | 24(1.6) | **0.04^c^** | 12(0.8) | 23(1.5) | 0.05^c^ |

Note:*P* values < 0.05 highlighted in bold.

^a^*P* values refer to differences in patients followed for 2 years and patients lost to follow-up

^b^*P* values refer to t-tests to evaluate mean differences of continuous data.

^c^*P* values refer to Chi-square tests for binary feature evaluation (feature true vs. feature not true).
